# Potential causal associations between leisure sedentary behaviors, physical activity, sleep traits, and myopia: a Mendelian randomization study

**DOI:** 10.1186/s12886-024-03367-z

**Published:** 2024-03-05

**Authors:** Xiao-Bin Zhang, He-He Jiang, Lin-Lin Zhang, Chun-Jing Li, Chen Chen, Meng-Zhen Xing, Yu-Ning Ma, Yu-Xia Ma

**Affiliations:** Shandong University of Traditional Chinese Medicine, Jinan, Shandong China

**Keywords:** Causal relationship, Myopia, Leisure sedentary behaviors, Physical activity, Sleep traits, Mendelian randomization analysis

## Abstract

**Background:**

Myopia is the most prevalent refractive error and a growing global health concern that significantly affects visual function. Researchers have recently emphasized considerably on the influence of lifestyle on myopia incidence and development. This study investigates the relationship between leisure sedentary behaviors (LSB)/physical activity (PA)/sleep traits and myopia.

**Methods:**

LSB, PA, and sleep trait-associated genetic variants were used as instrument variables in a Mendelian randomization (MR) study to examine their causal effects on myopia. Summary genome-wide association studies (GWASs) statistical data for LSB and PA were obtained from UK Biobank, and the data of sleep traits was obtained from UK Biobank, UK Biobank and 23andMe, and FinnGen. We used summary statistics data for myopia from MRC IEU. The MR analyses was performed using the inverse variance-weighted (IVW), MR-Egger, weighted median, and MR Pleiotropy RESidual Sum and Outlier methods.

**Results:**

Computer use was genetically predicted to increase the myopia risk [IVW odds ratio (OR) = 1.057; 95% confidence interval (CI), 1.038–1.078; *P* = 7.04 × 10^− 9^]. The self-reported moderate-to-vigorous physical activity (MVPA) (IVW OR = 0.962; 95% CI, 0.932–0.993; *P* = 1.57 × 10^− 2^) and television watching (IVW OR = 0.973; 95% CI, 0.961–0.985, *P* = 1.93 × 10^− 5^) were significantly associated with a lower myopia risk. However, genetically predicted sleep traits or accelerometer-measured physical activity had no significant associations with myopia.

**Conclusion:**

Our results indicated that computer use is a risk factor for myopia, whereas television watching and MVPA may protect against myopia. These findings shed new light on possible strategies for reducing the prevalence of myopia.

**Supplementary Information:**

The online version contains supplementary material available at 10.1186/s12886-024-03367-z.

## Introduction

Globally, myopia is the most common eye disease, with a prevalence of 22.9%. Its incidence is growing among young people, especially in Eastern and Southeast Asian countries, with 80–90% of their young people having myopia [1, 2]. The prevalence of this eye disease is high in East and Southeast Asia, led by China as well as in Europe and the United States, where myopia rates are increasing each year [3, 4]. In 2020, myopia affected 2,620 million people or 34% of the global population [5]. By 2050, it is expected to affect 4,758 million people or approximately half of the world’s population. Myopia, especially high myopia, can cause severe complications, such as glaucoma and vitreous clouding, and is a major cause of irreversible damage to eyesight [6, 7]. Myopia reduces the quality of life, restricts occupational options, negatively impacts the academic life and mental health of children, and imposes long-term health and economic burdens on society.

Many factors, both genetic and environmental, affect myopia onset and development [8]. Studies have recently focused more on the effect of lifestyle choices on disease incidence and progression. Leisure sedentary behaviors (LSB) refer to any awake behaviors involving an energy expenditure of < 1.5 metabolic equivalents in a reclining or seated position [9]. Adolescents who use electronic screens for > 6 h/day are approximately one times more likely to develop myopia than those who use them for < 2 h/day [10]. The relevant guidelines limit sedentary entertainment screen time for children and adolescents to no more than 2 h/day [11, 12]. Physical activity is among the most vital measures for preventing myopia [13]. Adequate daily outdoor activity time can decrease myopia prevalence in children and adolescents [14]. A study investigating 6,295 school-age children found that sleeping late is a risk factor for myopia [15].

Mendelian randomization (MR) is a statistical method for assessing the causal relationship between exposure factors and disease outcomes [16]. Genetic variations are employed as instrument variables (IVs) in the MR analysis because they are less susceptible to measurement error or bias. Additionally, because the disease cannot change the genotype, confounding variables and reverse causation can be minimized. The causal relationship between physical activity (PA), leisure sedentary behaviors (LSB), and sleep traits and myopia is unclear. At the same time, randomized controlled studies exploring this relationship by restricting participants’ PA, LSB, and sleep traits are impractical and unethical. We here describe this MR study to gain new insights into myopia pathogenesis.

## Methods

### Study design

**Fig. 1:**
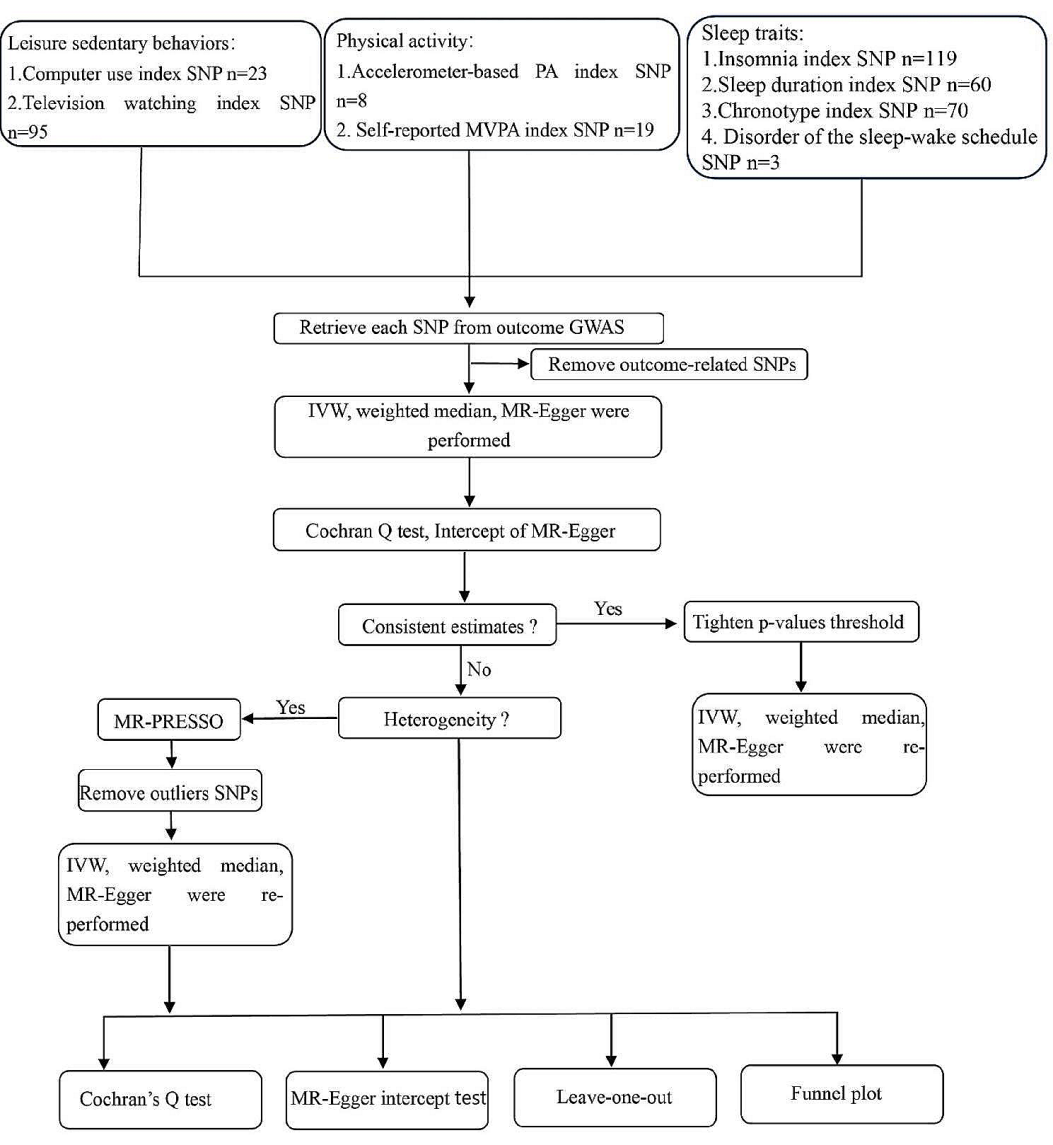
Workflow of Mendelian randomization study revealing causality from LSB, PA, and sleep traits on myopia

Using summary statistics from genome-wide association studies (GWASs), a two-sample MR analysis was performed to investigate the causal associations of PA/LSB/sleep traits with myopia. We performed linear MR analyses to estimate the associations between PA/LSB/sleep traits and myopia. To attain unbiased causal effects, the analysis must satisfy three assumptions: (1) genetic variants are strongly associated with the exposure of interest, (2) are not associated with potential confounders, and (3) influence outcomes only through the exposure of interest. Because this study included a re-analysis of gathered and published data, no additional ethical approval was required. Figure 1 presents the study design.

### Data sources for leisure sedentary behaviors, physical activity, and sleep-associated traits

The latest summary-level GWAS comprised 422,218 UK Biobank participants of European ancestry, and candidate genetic instruments for LSB were extracted from this study [17]. In the present GWAS meta-analyses, LSB primarily consisted of television watching, leisure computer use, and driving. Because the numbers of driving-related single-nucleotide polymorphisms (SNPs) were insufficient, they were not included in our investigation.

The PA-related summary statistics were obtained from the recently published GWAS, which was conducted using data of more than 377,000 participants of European descent from the UK Biobank [18]. We used two PA phenotypes, namely self-reported moderate-to-vigorous PA (MVPA) and accelerometer-assessed PA (average acceleration, APA). For moderate PA (MPA), the participants were asked the following question: “In a typical WEEK, on how many days did you do 10 min or more of moderate physical activities such as carrying light loads and cycling at a normal pace? (Do not include walking).” To assess vigorous PA (VPA), the participants were questioned as follows: “In a typical WEEK, how many days did you do 10 min or more of vigorous physical activities? (These are activities that make you sweat or breathe heavily such as fast cycling, aerobics, and heavy weight lifting).” MVPA was computed by taking the sum of total minutes/week of MPA multiplied by four and the sum of total minutes/week of VPA multiplied by eight, corresponding to their metabolic equivalents [18]. APA was measured using a wrist-worn Axivity AX3 accelerometer. In the invitation email and letter of device distribution shared with the participants, they were duly notified that the accelerometer had to be worn consistently and were permitted to proceed with their routine activities while wearing it. PA information (overall acceleration average) was extracted from 100 Hz raw triaxial acceleration data following wear/non-wear episode identification, calibration, and elimination of gravity and sensor noise. Individuals with data for < 3 days (72 h), those lacking data for each hour of the 24 h cycle, and outliers exhibiting means that deviated by more than four standard deviations were excluded from the study. The mean and standard deviation of the average acceleration were 27.98 and 8.14, respectively [19].

Genetic predictors for three sleep-related variables, namely insomnia, sleep duration, and chronotype, were obtained from the most recent GWAS. Using data of a GWAS involving 1,331,010 samples from the UK Biobank and 23andMe, the genetic association of insomnia was identified [20]. The estimated genetic association data for sleep duration were retrieved from the UK Biobank with 446,118 adults of European descent [21]. The estimated genetic association data for chronotype were retrieved from publicly available GWAS association data from the UK Biobank and 23andMe with 697,828 samples [22]. The analysis was conducted only using summary statistics obtained from the UK Biobank, encompassing 449,734 individuals of European descent. We used the data related to the sleep–wake schedule disorder (410 cases and 371,145 controls) obtained from the European samples of the FinnGen project (https://www.finngen.fi/en, accessed on September 21, 2023) [23]. Table S1 presents detailed information on the LSB, PA, and sleep-associated traits.

### Data source for myopia

460,536 participants of European ancestry were included in the analysis, which utilized myopia data integrated by the MRC IEU [Phenotype: Reason for glasses/contact lenses: For short-sightedness, i.e., only or mainly for distance viewing such as driving, cinema etc., (called ‘myopia’), GWAS ID “ukb-b-6353”] [24]. Brief information of the included traits are displayed in Table 1. Table S1 presents the specifics of all GWASs included in our study. The format and examples of UK Biobank, UK Biobank and 23andMe, MRC IEU data and linked data are shown in Table S2.

**Table 1:** Brief information of included traits in the MR analysis

Trait	Phenotype	Variable type	Participants	Ancestry
Leisure sedentary behaviors	Computer use	Exposure	422,218	European
Leisure sedentary behaviors	Television watching	Exposure	422,218	European
Physical activity	APA	Exposure	91,084	European
Physical activity	MVPA	Exposure	377,234	European
Validation-sleep traits	Insomnia	Exposure	1,331,010	European
Validation-sleep traits	Sleep duration	Exposure	446,118	European
Validation-sleep traits	Chronotype	Exposure	697,828	European
Validation-sleep patterns	Disorder of the sleep-wake schedule	Exposure	371,555	European
Myopia	Myopia	Outcome	460,536	European

### Selection of genetic instruments

We here used 10 sets of genetic instruments indicating LSB, PA, and sleep traits, namely (1) index SNPs representing leisure computer use (Table S3), (2) index SNPs representing leisure television watching (Table S4), (3) index SNPs representing APA (Table S5), (4) index SNPs representing MVPA (Table S6), (5) index SNPs representing insomnia (Table S7), (6) index SNPs representing sleep duration (Table S8), (7) index SNPs representing chronotype (Table S9), and (8) index SNPs representing the sleep–wake schedule disorder (Table S10).

We identified SNPs strongly associated with LSB/PA/insomnia/sleep duration/ chronotype so as to develop genetic instruments with statistically significant thresholds [*P* < 5 × 10^− 8^, linkage disequilibrium (LD) r^2^ < 0.001, LD distance > 10,000 kb]. SNPs associated with the sleep–wake schedule disorder were identified to develop genetic instruments with statistically significant thresholds [*P* < 5 × 10^− 7^, (LD) r^2^ < 0.01, LD distance > 10,000 kb] [25]. The F-statistic indicates the degree of association between SNPs and LSB/PA/sleep traits. SNPs with F > 10 are often believed to be highly likely to be linked to LSB/PA/sleep traits.

### MR analyses

After the effect alleles across the GWASs of LSB, PA, sleep traits, and myopia were harmonized, inverse-variance weighted (IVW), weighted median, and MR-Egger were used to determine MR estimates of LSB/PA/sleep traits for myopia. Regarding horizontal pleiotropy, these MR methods possess various underlying assumptions. The primary technique was IVW. IVW operates under the presumption that IVs may only affect the outcome through exposure [26]. In addition to IVW, the weighted median and MR-Egger methods were used in this study. When > 50% of the information originates from valid IVs, the weighted median method produces consistent estimates [27]. The MR-Egger method hypothesizes that variant–exposure associations are not related to the pleiotropic effects of genetic variants [27]. In our study, a tighter instrument *P* value criterion was established if the estimations made using these methods were inconsistent [28].

In MR studies, the sensitivity analysis is crucial for detecting underlying pleiotropy. Heterogeneity can be severely desecrated for MR estimates. In this study, potential horizontal pleiotropy was represented using heterogeneity markers (Cochran Q-derived *P* < 0.05) from the IVW method. The MR-Egger regression intercept indicated the presence of directional pleiotropy (*P* < 0.05) [29]. Moreover, the MR-Pleiotropy RESidual Sum and Outlier (MR-PRESSO) methods were applied to evaluate and correct horizontal pleiotropy [30]. The robustness of our findings was examined using the MR-Egger intercept, Cochran’s Q test, funnel pot, and leave-one-out analyses [31]. The intercept term from the MR-Egger regression was applied specifically to measure horizontal pleiotropy. Pleiotropy was judged to be present when *P* < 0.05. Heterogeneity was determined according to Cochran’s Q-test, for which the *P* value was 0.05. To determine whether a single SNP drove the causal association, we also conducted a leave-one-out analysis in which each exposure-related SNP was removed in turn, and the IVW analysis was repeated. The packages TwoSampleMR (version 0.5.7) and MR-PRESSO (version 1.0) in R (version 4.3.0) were used to perform the analyses.

## Results

All F-statistics for the IVs used for LSB were > 10 and ranged from 33.01 to 144.11. The median F-statistic was 39.13 and 43.37 for computer use and television watching, respectively, which suggested that weak instrument bias was impossible. Detailed data are presented in Tables S3 and S4. All F-statistics for the IVs used for PA were > 10 and ranged from 29.97 to 51.82. The median F-statistics for APA and MVPA were 31.04 and 31.1, respectively, which indicated that mild instrument bias was unlikely. Tables S5 and S6 present the detailed data. All F-statistics for the IVs used for insomnia, sleep duration, chronotype, and the sleep–wake schedule ranged from 22.56 to 441.00. The median F-statistics were 38.03, 35.05, 45.32, and 27.44 for insomnia, sleep duration, chronotype, and sleep–wake schedule disorder, respectively, which suggested that weak instrument bias was unlikely. The results were presented in Tables S7–S10.

### Causal effect from computer use to myopia

**Fig. 2:**
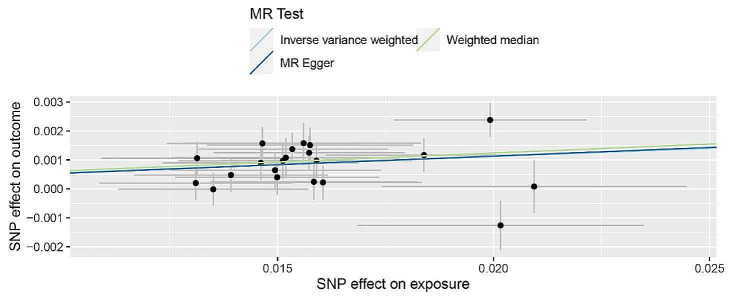
MR analysis of the causal effect of computer use on myopia

Following harmonization, which resulted in the removal of incompatible SNPs, 21 SNPs were found to be associated with computer use (rs631130, rs984409). The IVW method unveiled a substantial association between computer use and the increased myopia risk (OR [22] = 1.057; 95% CI, 1.038–1.078; *P* = 7.04 × 10^− 9^). Similarly, risk estimates were obtained using the weighted median (OR = 1.064; 95% CI, 1.038–1.091; *P* = 8.18 × 10^− 7^) and MR-Egger (OR = 1.061; 95% CI, 0.907–1.241;*P* = 4.66 × 10^− 1^) methods (Fig. 2). A Cochran Q-test-derived *P* value of 1.38 × 10^− 1^ for MR-Egger and a *P* value of 1.75 × 10^− 1^ for IVW indicated that heterogeneity was absent. Additionally, a significant intercept was not indicated (intercept = − 6.73 × 10^− 5^; SE = 1.26 × 10^− 3^; *P* = 9.58 × 10^− 1^), which showed that no directional pleiotropy was identified. A clear causal relationship was noted between computer use and myopia. According to the leave-one-out sensitivity analysis, no single SNP significantly deviated from the overall impact of computer use on myopia (Figure S1). Moreover, no pleiotropy was present, as exhibited by the funnel plot’s symmetry (Figure S2).

## Causal effect from television watching to myopia

**Fig. 3:**
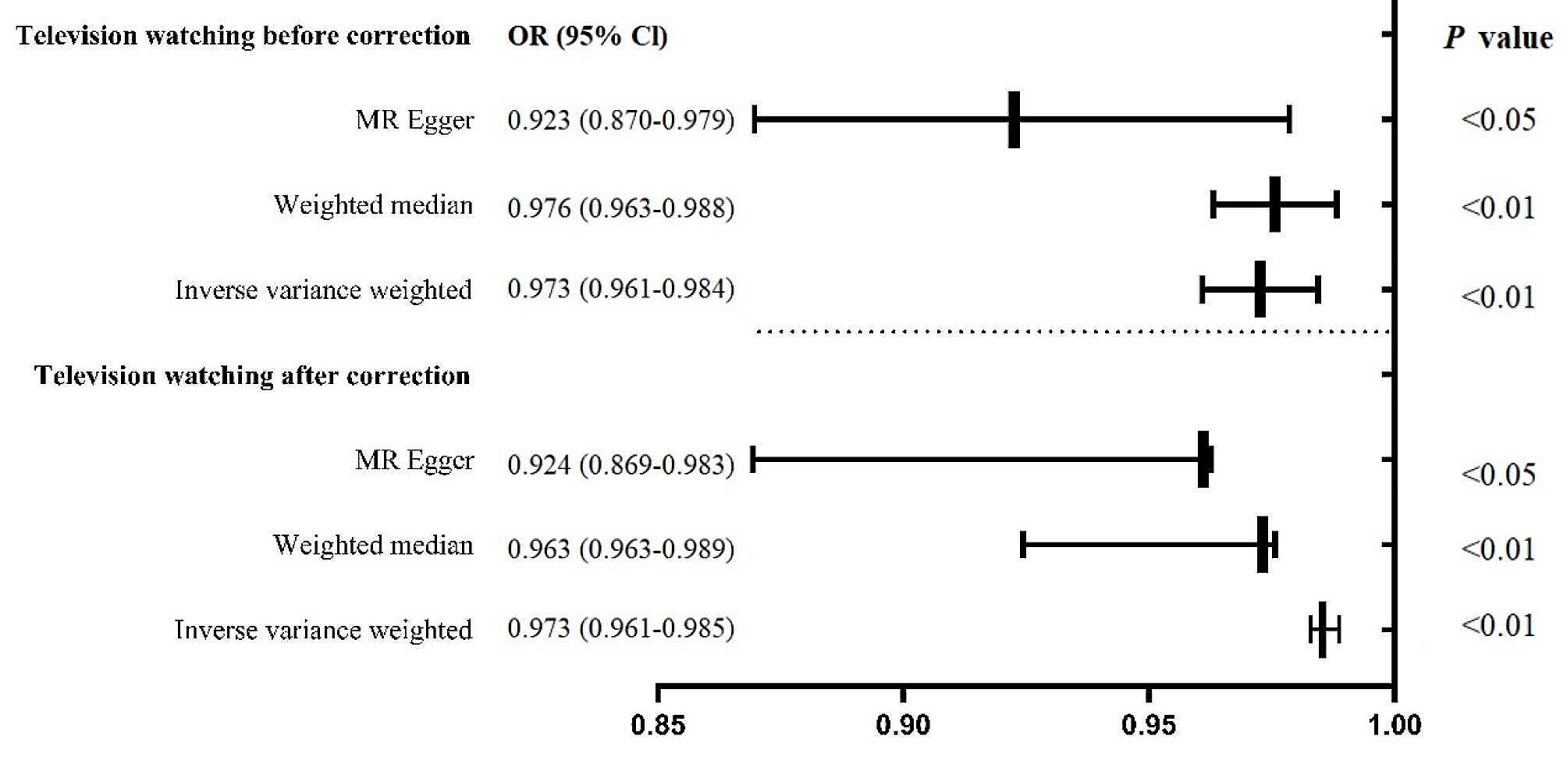
Odds ratio plot for television watching and myopia

After harmonization, 87 SNPs related to television watching were acquired to eliminate palindromic SNPs (rs17568389, rs61331678, rs62471080, rs7043521, and rs870151). Using the IVW approach, television watching was strongly linked to a lower myopia risk [odds ratio (OR) = 0.973; 95% confidence interval (CI), 0.961–0.984; *P* = 6.82 × 10^− 6^). Meanwhile, similar risk estimates were achieved using the MR-Egger method (OR = 0.923; 95% CI, 0.870–0.979; *P* = 8.90 × 10^− 3^) and weighted median (OR = 0.976; 95% CI, 0.963–0.988; *P* = 1.72 × 10^− 4^) methods. However, a Cochran Q-test-derived *P* value of 9.74 × 10^− 10^ for MR-Egger and a *P* value of 1.94 × 10^− 10^ for IVW indicated heterogeneity. MR-PRESSO also produced a comparable result (a global heterogeneity test *P* value of 2.00 × 10^− 4^). After three outliers (rs374722, rs7564130, and rs7693082) were excluded, the MR techniques were reapplied to assess the relationship between television watching and myopia (Fig. 3). On using the IVW method (OR = 0.973; 95% CI, 0.961–0.985; *P* = 1.93 × 10^− 5^), we noted that television watching substantively increased the myopia risk; similar risk estimates were obtained using the MR-Egger (OR = 0.924; 95% CI, 0.869–0.983; *P* = 1.42 × 10^− 2^) and weighted median (OR = 0.976; 95% CI, 0.963–0.989; *P* = 3.22 × 10^− 4^) methods. The MR estimates became substantial, thereby suggesting that a genetically predicted increase in television watching was considerably related to the myopia risk (Fig. 3). Figures S3 and S4 depict the MR regression slopes and individual causal estimates for each of the 84 SNPs. Furthermore, no proof of a significant intercept was noted (intercept = 8.70 × 10^− 4^; SE = 5.21 × 10^− 4^; *P* = 9.86 × 10^− 2^), which proved that no directional pleiotropy was observed. Indeed, television viewing was found to be causally related with myopia. In the leave-one-out sensitivity analysis, no single SNP significantly desecrated the aggregate effect of television viewing on myopia (Figure S5). In addition, the funnel plot was symmetrical, indicating that pleiotropy was absent (Figure S6).

### Causal effect from MVPA to myopia

**Fig. 4:**
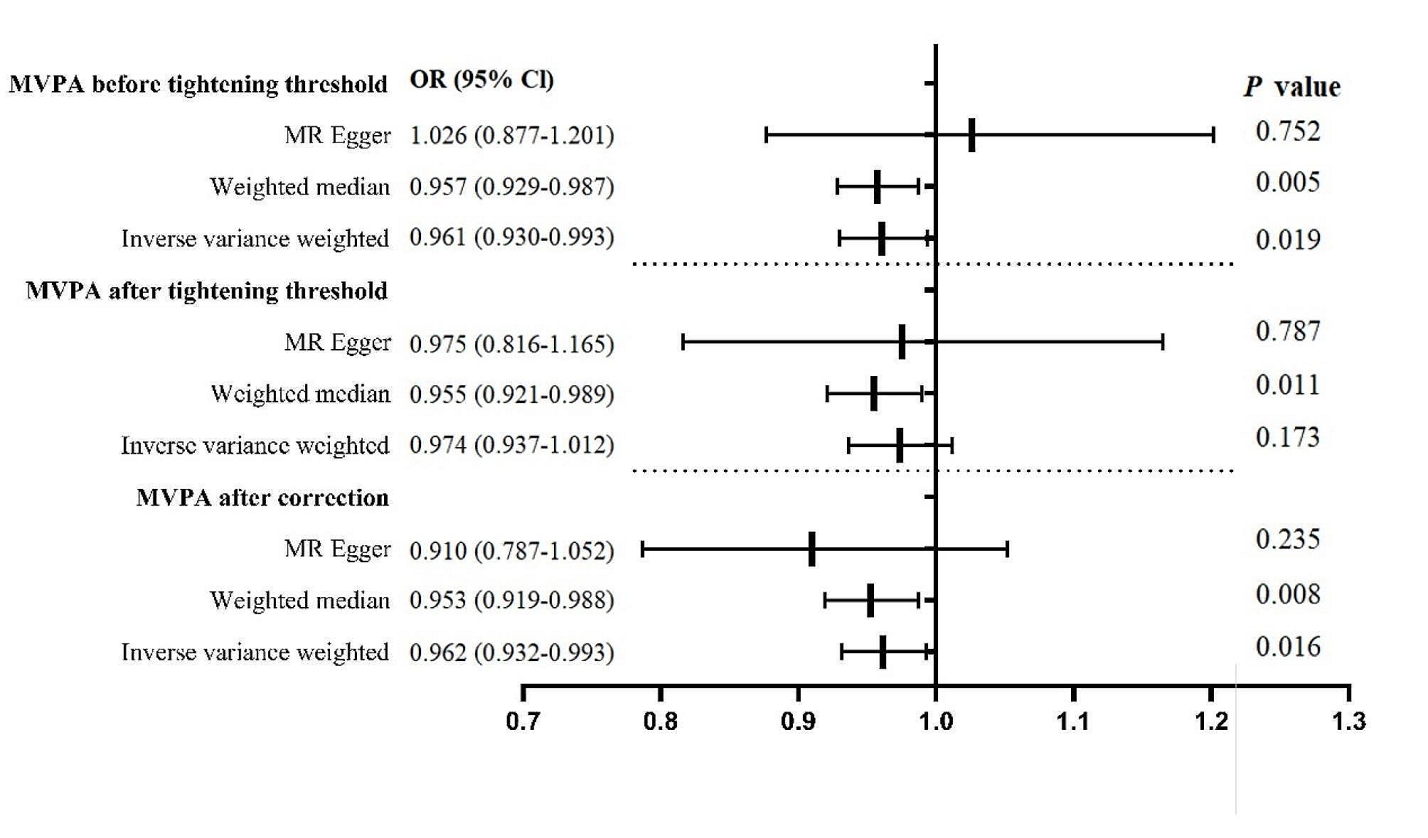
Odds ratio plot for MVPA and myopia

Using the 19 MVPA-related SNPs, the IVW (OR = 0.961; 95% CI, 0.930–0.993; *P* = 1.88 × 10^− 2^) and weighted median (OR = 0.957; 95% CI, 0.929–0.987; *P* = 5.09 × 10^− 3^) approaches unveiled that MVPA reduced the myopia risk significantly, whereas the MR-Egger approach (OR = 1.026; 95% CI, 0.877–−1.201; *P* = 7.52 × 10^− 1^) produced opposite results (Fig. 4). Because the results of the MR-Egger method of estimating MR were inconsistent with those of the weighted median and IVW methods, we tightened the instrument *P* value threshold to 3 × 10^− 8^ and used 12 SNPs as instrument tools [32]. The weighted median approach (OR = 0.955; 95% CI, 0.921–0.989; *P* = 1.12 × 10^− 2^) revealed a potential causal effect of MVPA on the myopia risk. By contrast, the IVW (OR = 0.974; 95% CI, 0.937–1.012; *P* = 1.73 × 10^− 1^) and MR-Egger regression (OR = 0.975; 95% CI, 0.816–1.165; *P* = 7.87 × 10^− 1^) methods revealed that MVPA had no significant association with the myopia risk (Fig. 4). The Cochran Q-test derived *P* values of 1.58 × 10^− 3^ and 2.85 × 10^− 3^ for MR-Egger and IVW, respectively, indicated heterogeneity. MR-PRESSO presented a different result (*P* value in the global heterogeneity test > 0.05). One outlier (rs2035562) removed from MVPA was detected using the MR-PRESSO test. The MR approaches were reapplied to evaluate the relationship between MVPA and myopia. Using the 11 MVPA-related SNPs, the IVW analysis showed that MVPA reduces the myopia risk (OR = 0.962; 95% CI, 0.932–0.993; *P* = 1.57 × 10^− 2^). Similar causal estimates were obtained from the weighted median approach (OR = 0.953; 95% CI, 0.919–0.988; *P* = 8.20 × 10^− 3^). The MR-Egger analysis revealed a consistent but nonsignificant direction (OR = 0.910; 95% CI, 0.787–1.052; *P* = 2.35 × 10^− 1^). The MR estimations became significant, demonstrating that a genetically predicted decrease in MVPA was linked with an increased myopia risk (Fig. 4). Figures S7 and S8 present the MR regression slopes and individual causal estimates of each of the 11 SNPs. Furthermore, no indication of a significant intercept was present (intercept = 8.97 × 10^− 4^; SE = 1.17 × 10^− 3^; *P* = 4.63 × 10^− 1^), demonstrating the absence of directional pleiotropy. In the leave-one-out sensitivity analysis, no one SNP was substantially violating the overall impact of MVPA on myopia (Figure S9). The funnel plot’s symmetry indicated a lack of pleiotropy (Figure S10).

### Causal effect from APA to myopia

Causal associations between APA and myopia were noted observed using the 8 APA-related SNPs. Table S11 summarizes the MR results.

### Causal effect from insomnia to myopia

In total, 119 index SNPs were selected to genetically predict insomnia. The findings revealed that insomnia had no causal relationship with myopia. Table S12 summarizes the MR results.

### Causal effect from sleep duration to myopia

A total of 60 index SNPs were selected to genetically predict sleep duration. According to the results, sleep duration had no causality on myopia. Table S13 presents the MR results.

### Causal effect from chronotype to myopia

In total, 170 index SNPs were selected to genetically predict chronotype. The results unveiled a lack of a causal connection between myopia and chronotype. The MR results are summarized in Table S14.

### Causal effect from the sleep–wake schedule disorder to myopia

A total of 3 index SNPs were acquired to genetically predict the sleep–wake schedule disorder. According to the findings, a lack of a causal association between myopia and the sleep–wake schedule disorder. Table S15 presents the MR results.

Among the sleep trait phenotypes, no causal associations were observed between genetically predicted sleep traits and myopia.

## Discussion

We here used three MR methods to examine the associations of genetically predicted LSB (computer use and television watching)/PA (MVPA and APA)/sleep traits (insomnia, sleep duration, chronotype, sleep–wake schedule disorder) with myopia. Our findings reveal that leisure television watching and MVPA may serve as preventative measures against myopia, but leisure computer use increases the myopia risk. Moreover, the relationship between APA/sleep traits and myopia was not supported by the evidence obtained.

Direct evidence of the causal relationships between LSB/PA/sleep traits and myopia is still lacking. Compared with the large-scale prospective clinical trials necessitating long-term observation, the MR study revealed the potential causal relationship between LSB/PA/sleep traits and myopia in a time- and cost-efficient manner.

We noted a correlation between computer-use and an increased risk of myopia. In support, a study on 5,074 children in Rotterdam revealed an association between increased computer-use and the development of myopia [33]. Similarly, increased computer-use among college students has been reported in correlation with an increase of myopia [34]. Another study found that working on a computer results in higher rates of myopia [35]. Thus, prolonged and intense computer-use elevates the likelihood of developing refractive disorders and visual fatigue [36]. Meanwhile, the accommodation latency is a significant contributor to myopia. An increased accommodation latency induced by proximity to computer-use may exacerbate the progression of myopia [37]. Consistent with these previous findings, our MR analyses unequivocally demonstrated a causal association between leisure computer-use and myopia. Notably, the present study showed that leisure television watching is a protective factor for myopia and that is reduces the risk of developing poor vision. A study on 240 Finnish children aged 8.7 to 12.8 years that was followed up for 23 years demonstrated that individuals who watched television for extended durations had reduced myopia rates [38]. The results of a longitudinal refractive study on 224 Norwegian students showed no association between refractive changes and time spent on watching television [39]. This observation may be attributed to the fact that television screens have become larger in recent years, and viewing the screen from afar has less impact on refractive change. The diopter hours (Dh) variable has quite commonly been used as a measure of near workload and as a risk factor for myopia [40]. When using Dh as a measure of near workload as a risk factor for myopia, for example, the Dh value obtained from 1 h spent solely on reading was the same as that obtained from spending 3 h solely on watching TV [41]. From these results, it can be deduced that watching television has a lesser impact on the risk of myopia. Nonetheless, watching TV is a more immersive and less reflective form of recreation than using a computer [42].

In a Poland study involving school children aged 9–11 years, higher PA levels positively affected the functional status in myopic children [43]. A recent study of 16- to 17-year-old adolescents revealed that PA protects against myopia [10]. By contrast, according to a recent meta-analysis, PA has no influence on myopia [44]. To address this inconsistency in results, we conducted MR estimations using two sets of genetic instruments. The findings unveiled that MVPA is associated with a lower myopia risk, which is consistent with the findings of previous studies. Most studies investigating the relationship between intraocular pressure (IOP) and exercise have reported that dynamic exercise duration is associated with IOP reduction [45]. The intensity of exercise also correlates with the magnitude of IOP reduction. Lower IOP facilitates in preventing myopia progression. Additionally, dynamic exercises cause alterations in ocular blood flow and an increase in local circulation [46]. Outdoor PA may inhibit myopia onset and progression. Numerous studies have attributed this phenomenon to exposure to brighter light, elevated dopamine levels, increased vitamin D levels, and UV light alone. Notably, no association was observed between APA and the myopia risk [47–49]. Estimates of PA can differ between self-reported values and objective measurements [50, 51]. Cognitive biases and affective states possibly influence self-report measures of PA; both of them impact the responses provided to self-reported questionnaires [52]. In the general adult population, self-reported MVPA took longer than APA [53]. PA data obtained from self-reported MVPA and APA differed conceptually, and these differences increased with activity and intensity levels [54]. This discrepancy regarding causality with myopia may be justified by these possibilities.

Our findings indicated no causality between the four sleep traits and myopia incidence, which is consistent with the results of several studies. For example, according to a prospective cohort study involving 1,194 adults, sleep quality in childhood is not associated with future development of myopia [55]. In a systematic review, the association between myopia and sleep duration or quality was also clinically nonsignificant [56]. Nonetheless, one study suggested that insufficient sleep duration increases the risk of eye disorders [57]. Circadian rhythm dysfunction is characterized by an irregular sleep–wake schedule [58]. During circadian rhythm disruption, the axial and choroidal daily rhythms change in phase, predisposing an individual to myopia [59]. Ciliary muscle inactivity during sleep and a reasonable sleep–wake schedule provide rest to the eyes and help in reducing the risk of eye diseases such as myopia [56, 60]. Therefore, regular and sufficient sleep are valuable for myopia prevention and treatment [61]. To further clarify the relationship between sleep traits and myopia, additional studies are warranted to supplement the present study findings in the future.

The MR study design is the primary strength of the present study. This design minimizes residual confounding variables and reverses causality inherent to observational studies. Moreover, it allows us to investigate the potential causality between LSB/PA/sleep traits and myopia. The IVs of LSB/PA/sleep traits included in this study had substantial sample sizes and had a robust association with focal exposure. Consequently, this mitigated the influence of weak instrument bias and increased the statistical power of the study. Furthermore, the consistency noted across the results of sensitivity analyses offers additional evidence for the validity of the effect estimates.

However, the limitations of this study must be acknowledged. First, because we used the data of participants of European ancestry, the findings are not directly applicable to other ethnic groups with distinct cultures and lifestyles. Next, our study congregated information on LSB, MVPA, and sleep traits from a self-reported questionnaire, as opposed to through objective measurements. This may be subject to information bias, which includes interviewee bias, interviewer bias, social desirability bias, recall bias, overestimation and underestimation of activities, and the potential for misclassification of activities [62]. Self-reported questionnaires are still undeniably a popular method of collecting data because of their following advantages: directly capture respondents’ true thoughts, low cost and ease of use, higher reliability, and greater suitability for large-scale studies [63, 64]. Furthermore, we could not execute sex- or age-specific analyses because we used summary statistics and individual raw measurements were not conducted. The myopia data used in this study do not have clearly stated myopia ranges, criteria, and measurements in databases at this stage, so specific analyses of the data in question could not be performed. The MR analysis is based on inferring causation from genetics; therefore, it can only clarify about potential causal linkages but cannot pinpoint the underlying specific biological route. In addition, other potential influencing factors in our research may have caused deviations, necessitating larger MR analyses.

## Conclusions

Our results suggest that computer use is causally related to the increased myopia risk, whereas television viewing and moderate physical activity may be causally linked to the decreased myopia risk. Our study offers new insights into the potential mechanism for predicting myopia occurrence and progression.

### Electronic supplementary material

Below is the link to the electronic supplementary material.

Supplementary Material 1

## Data Availability

The datasets analyzed in this study are summaries of publicly accessible statistics. Summary statistics of leisure sedentary behaviors including computer use and television watching were sourced from the UK Biobank (https://data.mendeley.com/datasets/mxjj6czsrd/1). The PA summary statistics were derived from a recently published GWAS conducted in the UK Biobank. Data on accelerometer-based physical activity measurement (average acceleration) was provided by the UK Biobank (GWAS ID ebi-a-GCST006099, https://gwas.mrcieu.ac.uk/datasets). Data on moderate to vigorous physical activity levels was provided by the UK Biobank (GWAS ID ebi-a-GCST006097, https://gwas.mrcieu.ac.uk/datasets). Data on insomnia was provided by the UK Biobank and 23andMe (available at https://ctg.cncr.nl/software/summary_statistics). Data on sleep duration was provided by the UK Biobank (available at https://sleep.hugeamp.org/downloads.html). Data on chronotype was provided by the UK Biobank (available at http://www.t2diabetesgenes.org/data/). Data on disorder of the sleep—wake schedule was provided by the FinnGen (available at https://storage.googleapis.com/finngen-public-data-r9/summary_stats/finngen_R9_F5_SLEEPWAKE.gz l). Summary statistics of myopia was obtained from the MRC Integrative Epidemiology Unit (IEU) OpenGWAS database (GWAS ID ukb-b-6353, https://gwas.mrcieu.ac.uk/datasets).
