# Supplementary material for: Potential causal associations between leisure sedentary behaviors, physical activity, sleep traits, and myopia: a Mendelian randomization study
Source: BMC Ophthalmol. 2024 Mar 5;24:104. doi: 10.1186/s12886-024-03367-z (PMC10913247; doi:10.1186/s12886-024-03367-z)
Supplement: Supplementary file 1 — Supplementary Material 1 [file 12886_2024_3367_MOESM1_ESM.doc]

**Supporting information**

**Potential Causal Association Between Leisure Sedentary Behaviors, Physical Activity, Sleep Traits and Myopia: A Mendelian Randomization Study**

Table S1. Details of the GWASs included in the Mendelian randomization

Table S2. The format and three examples of UK biobank, UK Biobank and 23andMe, MRC IEU data and linked data

Table S3. 23 index SNPs represented genetically predicted computer use

Table S4. 95 index SNPs represented genetically predicted television watching

Table S5. 8 index SNPs represented genetically predicted APA

Table S6. Table S5. 19 index SNPs represented genetically predicted self-reported MVPA

Table S7. 119 index SNPs represented genetically predicted insomnia

Table S8. 60 index SNPs represented genetically predicted sleep duration

Table S9. 170 index SNPs represented genetically predicted chronotype

Table S10. 3 index SNPs represented genetically predicted disorder of the sleep-wake schedule

Table S11. MR results of APA on myopia

Table S12. MR results of insomnia on myopia

Table S13. MR results of sleep duration on myopia

Table S14. MR results of chronotype on myopia

Table S15. MR results of disorder of the sleep-wake schedule on myopia

Figure S1. Leave-one-out analysis from genetically predicted computer use on myopia

Figure S2. Funnel plot from genetically predicted t computer use on myopia

Figure S3. Scatter plot of SNPs associated with television watching and their risk of myopia after outliers removal with MR-PRESSO.

Figure S4. Forest plot of SNPs associated with television watching and their risk of myopia after outliers removal with MR-PRESSO.

Figure S5. Leave-one-out of SNPs associated with television watching and their risk of myopia after outliers removal with MR-PRESSO.

Figure S6. Funnel plot of SNPs associated with television watching and their risk of myopia after outliers removal with MR-PRESSO.

Figure S7. Scatter plot of SNPs associated with MVPA and their risk of myopia after tightening instrument P value threshold and outliers removal with MR-PRESSO.

Figure S8. Forest plot of SNPs associated with MVPA and their risk of myopia after tightening instrument P value threshold and outliers removal with MR-PRESSO.

Figure S9. Funnel plot of SNPs associated with MVPA and their risk of myopia after tightening instrument P value threshold and outliers removal with MR-PRESSO.

Figure S10. Leave-one-out of SNPs associated with MVPA and their risk of myopia after tightening instrument P value threshold and outliers removal with MR-PRESSO.

**Table S1. Details of the GWASs included in the Mendelian randomization**

| **Exposure** | **Phenotype** | **Consortium** | **Participants** | **Ancestry** | **GWAS ID/PubMed ID Web source** |
| --- | --- | --- | --- | --- | --- |
| Leisure sedentary behaviors | Computer use | UK Biobank | 422,218 | European | <https://pubmed.ncbi.nlm.nih.gov/32317632/> |
| Leisure sedentary behaviors | Television watching | UK Biobank | 422,218 | European | https://pubmed.ncbi.nlm.nih.gov/32317632/ |
| Physical activity | APA | UK Biobank | 91,084 | European | https://gwas.mrcieu.ac.uk, PMID: 29899525 |
| Physical activity | MVPA | UK Biobank | 377,234 | European | [https://gwas.mrcieu.ac.uk](https://gwas.mrcieu.ac.uk/), PMID: 29899525 |
| Validation-sleep traits | Insomnia | UK Biobank and 23andMe | 1,331,010 | European | <https://ctg.cncr.nl/software/summary_statistics> |
| Validation-sleep traits | Sleep duration | UK Biobank | 446,118 | European | http://sleepdisordergenetics.org/ |
| Validation-sleep traits | Chronotype | UK Biobank and 23andMe | 697,828 | European | https://www.ebi.ac.uk/gwas/publications/30696823 |
| Validation-sleep patterns | Disorder of the sleep-wake schedule | FinnGen | 371555 | European | https://storage.googleapis.com/finngen-public-data-r9/summary_stats/finngen_R9_F5_SLEEPWAKE.gz |
| **Outcome** | **Phenotype** | **Consortium** | **Participants** | **Ancestry** | **GWAS ID/PubMed ID /Web source** |
| Myopia | Myopia | MRC IEU | 460,536 | European | https://gwas.mrcieu.ac.uk, GWAS ID “ukb-b-6353” |

**Table S2. The format and three examples of UK biobank, UK Biobank and 23andMe, MRC IEU data and linked data**

| **Exposure: Computer use** | | | | | | | | | | | | | | | | | | | |
| --- | --- | --- | --- | --- | --- | --- | --- | --- | --- | --- | --- | --- | --- | --- | --- | --- | --- | --- | --- |
| **uniqid** | **SNP** | **CHR** | **BP** | **GENPOS** | **ALLELE1** | **ALLELE0** | **A1FREQ** | | **INFO** | | **BETA** | | | | **SE** | | | **P_BOLT_LMM_INF** | |
| 1:10177_A_AC | rs367896724 | 1 | 10177 | 0 | A | AC | 0.60242 | | 0.467935 | | 0.0035504 | | | | 0.00328092 | | | 2.80E-01 | |
| 1:10352_T_TA | rs201106462 | 1 | 10352 | 0 | T | TA | 0.607385 | | 0.447895 | | 0.0038416 | | | | 0.00337591 | | | 2.60E-01 | |
| 1:10511_A_G | rs534229142 | 1 | 10511 | 0 | G | A | 0.998702 | | 0.438272 | | 0.0408662 | | | | 0.0456653 | | | 3.70E-01 | |
| **Exposure: Television watching** | | | | | | | | | | | | | | | | | | | |
| **uniqid** | **SNP** | **CHR** | **BP** | **GENPOS** | **ALLELE1** | **ALLELE0** | **A1FREQ** | | **INFO** | | **BETA** | | | **SE** | | | | **P_BOLT_LMM_INF** | |
| 1:11008_C_G | rs575272151 | 1 | 11008 | 0 | C | G | 0.913957 | | 0.495023 | | 0.0101923 | | | 0.00551347 | | | | 6.50E-02 | |
| 1:11012_C_G | rs544419019 | 1 | 11012 | 0 | C | G | 0.913957 | | 0.495023 | | 0.0101923 | | | 0.00551347 | | | | 6.50E-02 | |
| 1:13110_A_G | rs540538026 | 1 | 13110 | 0 | G | A | 0.94071 | | 0.391804 | | -0.0037795 | | | 0.00726942 | | | | 6.00E-01 | |
| **Exposure: APA** | | | | | | | | | | | | | | | | | | | |
| **#CHROM** | **POS** | **ID** | **REF** | **ALT** | **QUAL** | **FILTER** | **INFO** | | **FORMAT** | | | | **ebi-a-GCST006099** | | | | | | |
| 1 | 544584 | rs576404767 | C | T | . | PASS | AF=0.001841 | | ES:SE:LP:AF:ID | | | | -0.743473:0.591173:0.677781:0.001841:rs576404767 | | | | | | |
| 1 | 546697 | rs12025928 | A | G | . | PASS | AF=0.913283 | | ES:SE:LP:AF:ID | | | | 0.0243715:0.0891681:0.107905:0.913283:rs12025928 | | | | | | |
| 1 | 565111 | rs573042692 | T | C | . | PASS | AF=0.001552 | | ES:SE:LP:AF:ID | | | | 0.254981:0.730029:0.136677:0.001552:rs573042692 | | | | | | |
| **Exposure: MVPA** | | | | | | | | | | | | | | | | | | | |
| **#CHROM** | **POS** | **ID** | **REF** | **ALT** | **QUAL** | **FILTER** | **INFO** | | **FORMAT** | | | | **ebi-a-GCST006097** | | | | | | |
| 1 | 533198 | rs78497331 | C | T | . | PASS | AF=0.00103 | | ES:SE:LP:AF:ID | | | | 0.0441395:0.0587621:0.346787:0.00103:rs78497331 | | | | | | |
| 1 | 544584 | rs576404767 | C | T | . | PASS | AF=0.001844 | | ES:SE:LP:AF:ID | | | | -0.00197714:0.0359787:0.0177288:0.001844:rs576404767 | | | | | | |
| 1 | 546697 | rs12025928 | A | G | . | PASS | AF=0.913465 | | ES:SE:LP:AF:ID | | | | -0.00461201:0.00543171:0.39794:0.913465:rs12025928 | | | | | | |
| **Exposure: Insomnia** | | | | | | | | | | | | | | | | | | | |
| **SNP** | **UNIQUE_ID** | **CHR** | **BP** | **A1** | **A2** | **MAF** | **OR** | | **SE** | | | **P** | | | | | **N** | | **INFO** |
| rs12184267 | 1:715265:C_T | 1 | 715265 | T | C | 0.03316 | 0.9881 | | 0.01461 | | | 0.4146 | | | | | 381202 | | 0.926915 |
| rs12184277 | 1:715367:A_G | 1 | 715367 | G | A | 0.0333 | 0.9854 | | 0.01459 | | | 0.3135 | | | | | 381593 | | 0.931148 |
| rs12184279 | 1:717485:A_C | 1 | 717485 | A | C | 0.03329 | 0.9858 | | 0.01459 | | | 0.3253 | | | | | 381621 | | 0.92523 |
| **Exposure: Sleep duration** | | | | | | | | | | | | | | | | | | | |
| **SNP** | **CHR** | **BP** | **ALLELE1** | **ALLELE0** | **A1FREQ** | **INFO** | | **BETA_SLEEPDURATION** | | | | | **SE_SLEEPDURATION** | | | | | **P_SLEEPDURATION** | |
| rs2462492 | 1 | 54676 | C | T | 0.599285 | 0.340158 | | 0.00599948 | | | | | 0.00397896 | | | | | 0.13 | |
| rs3107975 | 1 | 55326 | T | C | 0.991605 | 0.324228 | | -0.00724454 | | | | | 0.0223127 | | | | | 0.71 | |
| 1:70728_C_T | 1 | 70728 | C | T | 0.997843 | 0.365713 | | -0.0575836 | | | | | 0.0402387 | | | | | 0.14 | |
| **Exposure: Chronotype** | | | | | | | | | | | | | | | | | | | |
| **CHR_ID** | **CHR_POS** | **REPORTED GENE(S)** | **MAPPED_GENE** | **SNP_GENE_IDS** | **STRONGEST SNP-RISK ALLELE** | **SNPS** | **SNP_ID_CURRENT** | | **RISK ALLELE FREQUENCY** | **P-VALUE** | | | **PVALUE_MLOG** | | | | **OR or BETA** | | **CHR_ID** |
| 12 | 22907429 | ETNK1, SOX5 | LINC02955 | ENSG00000256995 | rs2433634-C | rs2433634 | 2433634 | | 0.2759 | 5.00E-10 | | | 9.301029996 | | | | 1.0271623 | | 12 |
| 8 | 8410803 | SGK223, CLDN23 | PRAG1 - LINC02949 |  | rs2979139-G | rs2979139 | 2979139 | | 0.4952 | 4.00E-14 | | | 13.39794001 | | | | 1.0270597 | | 8 |
| 15 | 58677093 | ADAM10 | ADAM10 | ENSG00000137845 | rs4775086-G | rs4775086 | 4775086 | | 0.7637 | 1.00E-08 | | | 8 | | | | 1.0270597 | | 15 |
| **Exposure: Disorder of the sleep-wake schedule** | | | | | | | | | | | | | | | | | | | |
| **#chrom** | **pos** | **ref** | **alt** | **rsids** | **nearest_genes** | **pval** | **mlogp** | | **beta** | **sebeta** | | | **af_alt** | | | | **af_alt_cases** | | **af_alt_controls** |
| 1 | 13668 | G | A | rs2691328 | OR4F5 | 0.869208 | 0.0608765 | | 0.140779 | 0.854943 | | | 0.00584693 | | | | 0.00617408 | | 0.00584657 |
| 1 | 14773 | C | T | rs878915777 | OR4F5 | 0.291616 | 0.535188 | | 0.566572 | 0.537248 | | | 0.0134972 | | | | 0.0159732 | | 0.0134945 |
| 1 | 15585 | G | A | rs533630043 | OR4F5 | 0.444599 | 0.352032 | | 1.25226 | 1.63811 | | | 0.00111472 | | | | 0.00168819 | | 0.00111408 |
| **Outcome: Myopia** | | | | | | | | | | | | | | | | | | | |
| **#CHROM** | **POS** | **ID** | **REF** | **ALT** | **QUAL** | **FILTER** | **INFO** | | | **FORMAT** | | | | | | **UKB-b-6353** | | | |
| 1 | 49298 | rs10399793 | T | C | . | PASS | AF=0.623741 | | | ES:SE:LP:AF:ID | | | | | | 0.00055288:0.00100542:0.236572:0.623741:rs10399793 | | | |
| 1 | 54676 | rs2462492 | C | T | . | PASS | AF=0.400426 | | | ES:SE:LP:AF:ID | | | | | | -0.000768041:0.000996035:0.356547:0.400426:rs2462492 | | | |
| 1 | 86028 | rs114608975 | T | C | . | PASS | AF=0.103554 | | | ES:SE:LP:AF:ID | | | | | | 0.000971422:0.00159251:0.267606:0.103554:rs114608975 | | | |

**Table S3.23 index SNPs represented genetically predicted computer use**

| **SNP** | **Effect_allele** | **Other_allele** | **Beta** | **SE** | ***P*** | **EAF** | **R^2^** | **F-statistics** |
| --- | --- | --- | --- | --- | --- | --- | --- | --- |
| rs2748985 | T | C | -0.01534 | 0.002213 | 4.1E-12 | 0.454667 | 0.000114 | 48.04916 |
| rs6744254 | C | T | -0.0159 | 0.002204 | 5.4E-13 | 0.47126 | 0.000123 | 52.0438 |
| rs10208088 | C | T | 0.013103 | 0.002232 | 4.4E-09 | 0.418634 | 8.16E-05 | 34.46284 |
| rs11708955 | T | C | -0.01575 | 0.002387 | 4.2E-11 | 0.694765 | 0.000103 | 43.53657 |
| rs6774533 | C | T | -0.01494 | 0.002458 | 1.2E-09 | 0.294245 | 8.75E-05 | 36.94327 |
| rs2068625 | T | C | -0.01584 | 0.002398 | 4E-11 | 0.302374 | 0.000103 | 43.63248 |
| rs2220599 | C | G | -0.01605 | 0.00229 | 2.4E-12 | 0.632986 | 0.000116 | 49.12211 |
| rs11749912 | A | G | 0.01392 | 0.002237 | 4.9E-10 | 0.422554 | 9.17E-05 | 38.72081 |
| rs9372625 | G | A | -0.01839 | 0.002272 | 5.7E-16 | 0.61853 | 0.000155 | 65.5156 |
| rs55772938 | A | G | -0.01512 | 0.002417 | 3.9E-10 | 0.704749 | 9.27E-05 | 39.13346 |
| rs2345941 | A | G | 0.014647 | 0.002217 | 4E-11 | 0.550313 | 0.000103 | 43.64796 |
| rs13262595 | A | G | -0.01573 | 0.002215 | 1.2E-12 | 0.440345 | 0.000119 | 50.4322 |
| rs4977839 | G | A | -0.01992 | 0.002233 | 4.7E-19 | 0.584465 | 0.000188 | 79.57913 |
| rs113851275 | G | A | -0.02094 | 0.003537 | 3.2E-09 | 0.891576 | 8.3E-05 | 35.04945 |
| rs73578186 | C | T | 0.01499 | 0.002365 | 2.3E-10 | 0.675347 | 9.51E-05 | 40.17343 |
| rs2734849 | A | G | -0.01351 | 0.0022 | 8.2E-10 | 0.491453 | 8.93E-05 | 37.71059 |
| rs1448355 | C | T | -0.01461 | 0.002274 | 1.3E-10 | 0.381282 | 9.78E-05 | 41.27789 |
| rs35933007 | G | A | -0.01519 | 0.002644 | 9.2E-09 | 0.771615 | 7.82E-05 | 33.00582 |
| rs206965 | T | C | 0.015601 | 0.002715 | 9.1E-09 | 0.206729 | 7.82E-05 | 33.01894 |
| rs166835 | C | T | 0.013136 | 0.002222 | 3.4E-09 | 0.443342 | 8.28E-05 | 34.94911 |
| rs4073003 | A | G | 0.020163 | 0.00332 | 1.2E-09 | 0.873541 | 8.73E-05 | 36.8835 |
| rs984409 | A | T | -0.01409 | 0.002303 | 9.3E-10 | 0.358909 | 8.86E-05 | 37.43105 |
| rs631130 | A | G | 0.013109 | 0.002227 | 4E-09 | 0.435739 | 8.21E-05 | 34.64947 |

**Table S4. 95 index SNPs represented genetically predicted television watching**

| **SNP** | **Effect_allele** | **Other_allele** | **Beta** | **SE** | ***P*** | **EAF** | **R^2^** | **F-statistics** |
| --- | --- | --- | --- | --- | --- | --- | --- | --- |
| rs11810109 | A | T | 0.016252 | 0.002357 | 5.4E-12 | 0.701438 | 0.000113 | 47.54364 |
| rs984409 | G | A | -0.01487 | 0.002253 | 4.1E-11 | 0.363032 | 0.000103 | 43.56097 |
| rs17379561 | A | T | -0.02548 | 0.003072 | 1.1E-16 | 0.855876 | 0.000163 | 68.7946 |
| rs4845364 | A | G | -0.0153 | 0.002156 | 1.3E-12 | 0.495003 | 0.000119 | 50.35972 |
| rs6673341 | T | G | -0.01451 | 0.002169 | 2.2E-11 | 0.465333 | 0.000106 | 44.75213 |
| rs10737620 | T | A | 0.014414 | 0.002419 | 2.6E-09 | 0.273668 | 8.41E-05 | 35.50547 |
| rs6721975 | T | C | -0.01669 | 0.002614 | 1.7E-10 | 0.231524 | 9.65E-05 | 40.76613 |
| rs72781699 | G | A | -0.01868 | 0.002678 | 3E-12 | 0.797367 | 0.000115 | 48.65534 |
| rs12105701 | C | T | -0.01288 | 0.002208 | 5.4E-09 | 0.395851 | 8.06E-05 | 34.02762 |
| rs7564130 | T | C | -0.01499 | 0.002251 | 2.8E-11 | 0.640601 | 0.000105 | 44.34557 |
| rs10189857 | A | G | -0.02046 | 0.00218 | 6.2E-21 | 0.56785 | 0.000209 | 88.08383 |
| rs62641636 | A | G | 0.014424 | 0.002339 | 7E-10 | 0.691839 | 9.01E-05 | 38.02848 |
| rs11689199 | A | G | 0.018464 | 0.002204 | 5.5E-17 | 0.597665 | 0.000166 | 70.1821 |
| rs1451533 | G | A | -0.0157 | 0.002433 | 1.1E-10 | 0.724835 | 9.86E-05 | 41.64022 |
| rs374722 | G | A | 0.024493 | 0.003024 | 5.5E-16 | 0.150476 | 0.000155 | 65.60219 |
| rs3754970 | T | C | -0.015 | 0.00217 | 4.8E-12 | 0.495822 | 0.000113 | 47.78163 |
| rs4577309 | A | G | 0.016004 | 0.002168 | 1.6E-13 | 0.468698 | 0.000129 | 54.49247 |
| rs10932837 | C | T | -0.01311 | 0.002162 | 1.3E-09 | 0.494411 | 8.71E-05 | 36.76986 |
| rs4973576 | C | A | -0.01452 | 0.002379 | 1E-09 | 0.298072 | 8.82E-05 | 37.25137 |
| rs9834970 | T | C | 0.012768 | 0.002158 | 3.3E-09 | 0.501031 | 8.29E-05 | 35.00586 |
| rs3796386 | G | A | -0.02617 | 0.00218 | 3.2E-33 | 0.571888 | 0.000341 | 144.1094 |
| rs11130793 | C | T | 0.012922 | 0.002211 | 5E-09 | 0.601737 | 8.09E-05 | 34.15702 |
| rs11714337 | G | A | 0.014402 | 0.002189 | 4.7E-11 | 0.569041 | 0.000103 | 43.28646 |
| rs6797840 | A | C | -0.0161 | 0.002184 | 1.7E-13 | 0.455899 | 0.000129 | 54.3431 |
| rs2034768 | A | G | 0.014736 | 0.002158 | 8.6E-12 | 0.48743 | 0.00011 | 46.62881 |
| rs9867121 | C | A | 0.019499 | 0.002809 | 3.9E-12 | 0.817728 | 0.000114 | 48.18581 |
| rs12491503 | G | A | -0.01426 | 0.002299 | 5.5E-10 | 0.670306 | 9.11E-05 | 38.47327 |
| rs114600294 | G | C | -0.01624 | 0.002642 | 7.9E-10 | 0.788982 | 8.95E-05 | 37.78369 |
| rs66852340 | C | T | -0.01779 | 0.002601 | 7.9E-12 | 0.775527 | 0.000111 | 46.78096 |
| rs34811474 | G | A | 0.015283 | 0.002557 | 2.3E-09 | 0.767813 | 8.46E-05 | 35.72348 |
| rs6850494 | A | C | -0.01433 | 0.002222 | 1.1E-10 | 0.615571 | 9.85E-05 | 41.59127 |
| rs13107325 | C | T | -0.02917 | 0.004123 | 1.5E-12 | 0.925644 | 0.000119 | 50.05462 |
| rs7693703 | G | A | 0.022844 | 0.003841 | 2.7E-09 | 0.910033 | 8.38E-05 | 35.37151 |
| rs6825241 | C | A | -0.01696 | 0.002166 | 4.9E-15 | 0.536204 | 0.000145 | 61.3102 |
| rs7693082 | G | C | 0.015072 | 0.002358 | 1.7E-10 | 0.299686 | 9.68E-05 | 40.85565 |
| rs262890 | A | G | -0.01857 | 0.002356 | 3.2E-15 | 0.698905 | 0.000147 | 62.12568 |
| rs7716447 | A | G | -0.01338 | 0.002269 | 3.6E-09 | 0.639406 | 8.24E-05 | 34.77292 |
| rs1031423 | T | C | -0.01852 | 0.002626 | 1.8E-12 | 0.215208 | 0.000118 | 49.73825 |
| rs57585211 | T | G | -0.01673 | 0.00285 | 4.3E-09 | 0.826832 | 8.16E-05 | 34.4588 |
| rs10041724 | T | C | 0.018093 | 0.002739 | 3.9E-11 | 0.807695 | 0.000103 | 43.63501 |
| rs62379379 | G | T | -0.02602 | 0.004232 | 7.8E-10 | 0.928551 | 8.95E-05 | 37.80256 |
| rs10054327 | G | A | 0.017245 | 0.00219 | 3.4E-15 | 0.576024 | 0.000147 | 62.00634 |
| rs42210 | G | C | -0.01385 | 0.002395 | 7.3E-09 | 0.289169 | 7.92E-05 | 33.44155 |
| rs72828890 | C | T | 0.019292 | 0.003298 | 4.9E-09 | 0.869256 | 8.1E-05 | 34.21773 |
| rs72834698 | G | A | 0.022656 | 0.003101 | 2.7E-13 | 0.858074 | 0.000126 | 53.37783 |
| rs9471333 | C | T | 0.013104 | 0.002169 | 1.5E-09 | 0.450187 | 8.64E-05 | 36.49947 |
| rs6905544 | A | G | -0.01899 | 0.00221 | 8.5E-18 | 0.398622 | 0.000175 | 73.83518 |
| rs17789218 | T | C | 0.018597 | 0.002514 | 1.4E-13 | 0.75605 | 0.00013 | 54.72089 |
| rs2184364 | A | G | 0.01564 | 0.002637 | 3E-09 | 0.782382 | 8.33E-05 | 35.17638 |
| rs9718104 | T | G | -0.04077 | 0.00461 | 9.3E-19 | 0.94155 | 0.000185 | 78.21274 |
| rs17568389 | T | A | 0.015481 | 0.002165 | 8.6E-13 | 0.486903 | 0.000121 | 51.13051 |
| rs62471080 | G | C | -0.01278 | 0.002167 | 3.7E-09 | 0.540782 | 8.24E-05 | 34.78098 |
| rs7834121 | G | T | -0.01396 | 0.002164 | 1.1E-10 | 0.504369 | 9.86E-05 | 41.61542 |
| rs6472942 | T | C | -0.01316 | 0.002188 | 1.8E-09 | 0.568486 | 8.57E-05 | 36.17555 |
| rs2616830 | G | A | 0.016465 | 0.002166 | 2.9E-14 | 0.461988 | 0.000137 | 57.78359 |
| rs34864022 | A | G | -0.02638 | 0.004357 | 1.4E-09 | 0.933558 | 8.68E-05 | 36.65831 |
| rs12554512 | T | C | 0.020702 | 0.002193 | 3.8E-21 | 0.584071 | 0.000211 | 89.11387 |
| rs870151 | T | A | -0.01563 | 0.002187 | 8.8E-13 | 0.527632 | 0.000121 | 51.07617 |
| rs7043521 | A | T | 0.01427 | 0.002185 | 6.5E-11 | 0.430653 | 0.000101 | 42.65235 |
| rs4382592 | T | G | 0.013672 | 0.002358 | 6.7E-09 | 0.301425 | 7.96E-05 | 33.6182 |
| rs2073869 | C | T | 0.018611 | 0.002908 | 1.5E-10 | 0.833626 | 9.7E-05 | 40.95893 |
| rs1243182 | C | T | -0.01859 | 0.002342 | 2E-15 | 0.689639 | 0.000149 | 63.00615 |
| rs2045147 | A | G | 0.012669 | 0.002177 | 5.9E-09 | 0.449407 | 8.02E-05 | 33.86614 |
| rs10786658 | A | T | -0.01445 | 0.002196 | 4.6E-11 | 0.413503 | 0.000103 | 43.29811 |
| rs11245482 | T | C | -0.01323 | 0.002221 | 2.6E-09 | 0.61325 | 8.4E-05 | 35.48301 |
| rs17727474 | C | T | 0.017551 | 0.002961 | 3.1E-09 | 0.832295 | 8.32E-05 | 35.13378 |
| rs801733 | A | C | 0.016859 | 0.002253 | 7.3E-14 | 0.641215 | 0.000133 | 55.9937 |
| rs17207890 | G | A | 0.015694 | 0.002287 | 6.7E-12 | 0.657407 | 0.000112 | 47.09046 |
| rs11218575 | C | T | 0.015375 | 0.002187 | 2.1E-12 | 0.566037 | 0.000117 | 49.42317 |
| rs10772643 | C | T | 0.0248 | 0.003497 | 1.3E-12 | 0.107916 | 0.000119 | 50.29329 |
| rs10771746 | C | T | -0.01428 | 0.002395 | 2.5E-09 | 0.716605 | 8.42E-05 | 35.5503 |
| rs10876864 | G | A | -0.01334 | 0.002184 | 1E-09 | 0.427197 | 8.84E-05 | 37.3082 |
| rs8756 | C | A | -0.01345 | 0.002166 | 5.3E-10 | 0.484619 | 9.13E-05 | 38.55899 |
| rs2173650 | G | T | 0.017845 | 0.003044 | 4.6E-09 | 0.852206 | 8.14E-05 | 34.36701 |
| rs9563168 | G | A | 0.017587 | 0.002667 | 4.3E-11 | 0.791482 | 0.000103 | 43.4846 |
| rs9569734 | A | G | 0.018885 | 0.003007 | 3.4E-10 | 0.843521 | 9.34E-05 | 39.44256 |
| rs56858768 | G | A | -0.01488 | 0.00237 | 3.4E-10 | 0.700762 | 9.34E-05 | 39.41914 |
| rs7991062 | C | G | -0.01774 | 0.002287 | 8.8E-15 | 0.658663 | 0.000142 | 60.16901 |
| rs10145592 | C | G | -0.01485 | 0.002208 | 1.8E-11 | 0.409418 | 0.000107 | 45.23272 |
| rs2460 | G | A | -0.01529 | 0.002459 | 5E-10 | 0.736281 | 9.16E-05 | 38.66303 |
| rs61331678 | G | C | 0.015046 | 0.002187 | 6.1E-12 | 0.567932 | 0.000112 | 47.33065 |
| rs7189927 | T | C | 0.014974 | 0.002259 | 3.4E-11 | 0.355786 | 0.000104 | 43.93809 |
| rs749671 | G | A | 0.015654 | 0.002238 | 2.7E-12 | 0.62863 | 0.000116 | 48.92473 |
| rs7184800 | G | A | 0.016817 | 0.002349 | 8.2E-13 | 0.696784 | 0.000121 | 51.25414 |
| rs142710267 | T | G | 0.016245 | 0.002366 | 6.6E-12 | 0.65012 | 0.000112 | 47.14199 |
| rs2447098 | C | A | -0.01495 | 0.002178 | 6.8E-12 | 0.475175 | 0.000112 | 47.11558 |
| rs2584597 | T | C | 0.0151 | 0.002396 | 2.9E-10 | 0.662057 | 9.41E-05 | 39.71716 |
| rs9902312 | T | C | 0.015324 | 0.002327 | 4.5E-11 | 0.683148 | 0.000103 | 43.36601 |
| rs303753 | G | A | -0.01446 | 0.002291 | 2.7E-10 | 0.652948 | 9.43E-05 | 39.83679 |
| rs9964724 | C | T | 0.017647 | 0.002327 | 3.3E-14 | 0.317881 | 0.000136 | 57.51047 |
| rs7248205 | C | T | 0.013925 | 0.002217 | 3.4E-10 | 0.397614 | 9.34E-05 | 39.4509 |
| rs111901094 | G | T | -0.01708 | 0.002847 | 2E-09 | 0.817212 | 8.52E-05 | 35.9914 |
| rs6131281 | C | T | 0.016081 | 0.002206 | 3.1E-13 | 0.596509 | 0.000126 | 53.13896 |
| rs6141814 | C | A | -0.0135 | 0.002226 | 1.3E-09 | 0.613455 | 8.71E-05 | 36.78029 |
| rs56103247 | C | T | 0.029765 | 0.004752 | 3.8E-10 | 0.943942 | 9.29E-05 | 39.23348 |

**Table S5. 8 index SNPs represented genetically predicted APA**

| **SNP** | **Effect_allele** | **Other_allele** | **Beta** | **SE** | ***P*** | **EAF** | **R^2^** | **F-statistics** |
| --- | --- | --- | --- | --- | --- | --- | --- | --- |
| rs34517439 | A | C | -0.30792 | 0.056244 | 4.4E-08 | 0.120938 | 0.000329 | 29.97155 |
| rs6775319 | T | A | -0.22508 | 0.040802 | 3.5E-08 | 0.729248 | 0.000334 | 30.43023 |
| rs9293503 | C | T | -0.32895 | 0.058685 | 2.1E-08 | 0.111505 | 0.000345 | 31.41962 |
| rs12522261 | A | G | -0.21052 | 0.038304 | 3.9E-08 | 0.343473 | 0.000332 | 30.20659 |
| rs11012732 | G | A | -0.22499 | 0.038564 | 5.4E-09 | 0.332193 | 0.000374 | 34.03573 |
| rs148193266 | C | A | 0.510362 | 0.092154 | 3.1E-08 | 0.042668 | 0.000337 | 30.67008 |
| rs56194509 | G | T | 0.303404 | 0.043941 | 5E-12 | 0.220486 | 0.000523 | 47.67571 |
| rs59499656 | T | A | 0.228253 | 0.038256 | 2.4E-09 | 0.344493 | 0.000391 | 35.59734 |

**Table S6. Table S5. 19 index SNPs represented genetically predicted self-reported MVPA**

| **SNP** | **Effect_allele** | **Other_allele** | **Beta** | **SE** | ***P*** | **EAF** | **R^2^** | **F-statistics** |
| --- | --- | --- | --- | --- | --- | --- | --- | --- |
| rs2942127 | A | G | -0.01604 | 0.002903 | 3.3E-08 | 0.824644 | 8.09E-05 | 30.53361 |
| rs1974771 | A | G | 0.021339 | 0.003678 | 6.6E-09 | 0.099975 | 8.92E-05 | 33.65405 |
| rs2114286 | G | A | 0.012245 | 0.002217 | 3.3E-08 | 0.534243 | 8.08E-05 | 30.49902 |
| rs877483 | C | T | -0.01223 | 0.002228 | 4E-08 | 0.566815 | 7.99E-05 | 30.14337 |
| rs2035562 | G | A | 0.013876 | 0.002356 | 3.9E-09 | 0.672483 | 9.19E-05 | 34.68595 |
| rs1972763 | T | C | -0.01284 | 0.002324 | 3.3E-08 | 0.657628 | 8.09E-05 | 30.53392 |
| rs77742115 | C | T | 0.018348 | 0.003198 | 9.6E-09 | 0.138319 | 8.73E-05 | 32.92158 |
| rs2854277 | T | C | -0.03203 | 0.005067 | 2.6E-10 | 0.082571 | 0.000106 | 39.96234 |
| rs7804463 | C | T | -0.01501 | 0.002213 | 1.2E-11 | 0.470424 | 0.000122 | 45.99035 |
| rs921915 | C | T | 0.013888 | 0.00224 | 5.7E-10 | 0.587905 | 0.000102 | 38.43533 |
| rs1186721 | A | G | 0.01299 | 0.002372 | 4.4E-08 | 0.315844 | 7.95E-05 | 29.98413 |
| rs1043595 | A | G | -0.01441 | 0.002454 | 4.3E-09 | 0.282865 | 9.14E-05 | 34.47624 |
| rs2988004 | G | T | 0.013171 | 0.00224 | 4.1E-09 | 0.442245 | 9.17E-05 | 34.57965 |
| rs7326482 | T | G | 0.012961 | 0.002294 | 1.6E-08 | 0.615163 | 8.46E-05 | 31.91739 |
| rs10145335 | A | G | 0.014122 | 0.002541 | 2.7E-08 | 0.250611 | 8.18E-05 | 30.87788 |
| rs12912808 | T | C | -0.01755 | 0.003109 | 1.7E-08 | 0.148607 | 8.45E-05 | 31.867 |
| rs4886868 | G | T | 0.012495 | 0.002266 | 3.5E-08 | 0.585862 | 8.06E-05 | 30.40243 |
| rs429358 | C | T | 0.021982 | 0.003054 | 6.1E-13 | 0.154172 | 0.000137 | 51.8226 |
| rs1921981 | A | G | -0.01304 | 0.002371 | 3.8E-08 | 0.325647 | 8.02E-05 | 30.23757 |

**Table S7. 119 index SNPs represented genetically predicted insomnia**

| **SNP** | **Effect_allele** | **Other_allele** | **Beta** | **SE** | ***P*** | **EAF** | **R^2^** | **F-statistics** |
| --- | --- | --- | --- | --- | --- | --- | --- | --- |
| rs1031654 | A | C | -0.051 | 0.007 | 3.20E-13 | 0.798844 | 3.988E-05 | 53.08155 |
| rs10756571 | T | C | 0.036 | 0.006 | 1.97E-09 | 0.681717 | 2.705E-05 | 35.99995 |
| rs10758593 | A | G | -0.036 | 0.006 | 1.97E-09 | 0.399171 | 2.705E-05 | 35.99995 |
| rs10761240 | A | G | -0.043 | 0.006 | 7.68E-13 | 0.395628 | 3.859E-05 | 51.36103 |
| rs10800992 | T | C | 0.042 | 0.006 | 2.56E-12 | 0.44306 | 3.681E-05 | 48.99993 |
| rs10865954 | T | C | 0.042 | 0.006 | 2.56E-12 | 0.665632 | 3.681E-05 | 48.99993 |
| rs10898940 | A | C | 0.034 | 0.006 | 1.46E-08 | 0.482983 | 2.412E-05 | 32.11106 |
| rs10944696 | A | G | -0.038 | 0.007 | 5.68E-08 | 0.299156 | 2.214E-05 | 29.46934 |
| rs10947987 | T | C | -0.033 | 0.006 | 3.80E-08 | 0.443021 | 2.273E-05 | 30.24995 |
| rs10955647 | T | G | 0.033 | 0.006 | 3.80E-08 | 0.53224 | 2.273E-05 | 30.24995 |
| rs11001276 | A | T | -0.038 | 0.007 | 5.68E-08 | 0.261463 | 2.214E-05 | 29.46934 |
| rs11119409 | T | C | -0.035 | 0.006 | 5.43E-09 | 0.585949 | 2.556E-05 | 34.02773 |
| rs11149313 | A | G | 0.04 | 0.007 | 1.10E-08 | 0.272346 | 2.453E-05 | 32.65301 |
| rs11588755 | A | G | -0.035 | 0.006 | 5.43E-09 | 0.521703 | 2.556E-05 | 34.02773 |
| rs11605348 | A | G | -0.045 | 0.006 | 6.38E-14 | 0.349814 | 4.226E-05 | 56.24992 |
| rs116466468 | T | C | 0.044 | 0.007 | 3.26E-10 | 0.241146 | 2.968E-05 | 39.51014 |
| rs11722569 | T | C | 0.034 | 0.006 | 1.46E-08 | 0.657819 | 2.412E-05 | 32.11106 |
| rs11756035 | C | G | 0.051 | 0.009 | 1.46E-08 | 0.133031 | 2.412E-05 | 32.11106 |
| rs118166957 | T | C | 0.068 | 0.008 | 1.90E-17 | 0.164058 | 5.428E-05 | 72.24989 |
| rs12030482 | A | T | 0.041 | 0.007 | 4.71E-09 | 0.220864 | 2.577E-05 | 34.30607 |
| rs12310246 | A | G | 0.045 | 0.007 | 1.29E-10 | 0.249045 | 3.105E-05 | 41.32647 |
| rs12666306 | A | G | 0.042 | 0.006 | 2.56E-12 | 0.498933 | 3.681E-05 | 48.99993 |
| rs1289939 | T | C | -0.041 | 0.007 | 4.71E-09 | 0.76742 | 2.577E-05 | 34.30607 |
| rs12912299 | T | C | -0.043 | 0.006 | 7.68E-13 | 0.489008 | 3.859E-05 | 51.36103 |
| rs12917449 | A | C | -0.042 | 0.008 | 1.52E-07 | 0.195803 | 2.071E-05 | 27.56246 |
| rs12983032 | A | G | -0.043 | 0.006 | 7.68E-13 | 0.343392 | 3.859E-05 | 51.36103 |
| rs12991815 | C | G | 0.04 | 0.006 | 2.62E-11 | 0.575798 | 3.339E-05 | 44.44438 |
| rs13138995 | A | G | 0.034 | 0.006 | 1.46E-08 | 0.608071 | 2.412E-05 | 32.11106 |
| rs1530938 | A | G | 0.036 | 0.006 | 1.97E-09 | 0.557257 | 2.705E-05 | 35.99995 |
| rs1567084 | A | G | 0.033 | 0.006 | 3.80E-08 | 0.50127 | 2.273E-05 | 30.24995 |
| rs16903122 | T | C | 0.055 | 0.007 | 3.93E-15 | 0.249011 | 4.638E-05 | 61.7346 |
| rs16990210 | T | C | -0.046 | 0.008 | 8.92E-09 | 0.152726 | 2.484E-05 | 33.06245 |
| rs17025198 | A | G | 0.041 | 0.007 | 4.71E-09 | 0.205383 | 2.577E-05 | 34.30607 |
| rs17083297 | A | C | -0.044 | 0.008 | 3.80E-08 | 0.176432 | 2.273E-05 | 30.24995 |
| rs17223714 | A | G | 0.046 | 0.007 | 4.98E-11 | 0.212258 | 3.244E-05 | 43.18361 |
| rs17367725 | T | C | -0.036 | 0.006 | 1.97E-09 | 0.351718 | 2.705E-05 | 35.99995 |
| rs17520265 | A | G | -0.091 | 0.016 | 1.29E-08 | 0.037823 | 2.43E-05 | 32.34761 |
| rs17643634 | T | C | -0.06 | 0.008 | 6.38E-14 | 0.168918 | 4.226E-05 | 56.24992 |
| rs190073 | A | G | -0.034 | 0.006 | 1.46E-08 | 0.414422 | 2.412E-05 | 32.11106 |
| rs1927902 | T | C | 0.053 | 0.007 | 3.69E-14 | 0.745393 | 4.307E-05 | 57.32644 |
| rs2089358 | T | C | -0.041 | 0.007 | 4.71E-09 | 0.701186 | 2.577E-05 | 34.30607 |
| rs214934 | A | T | -0.038 | 0.006 | 2.40E-10 | 0.312241 | 3.013E-05 | 40.11105 |
| rs2216427 | C | G | 0.035 | 0.006 | 5.43E-09 | 0.347714 | 2.556E-05 | 34.02773 |
| rs2221119 | C | G | 0.036 | 0.006 | 1.97E-09 | 0.443685 | 2.705E-05 | 35.99995 |
| rs224029 | T | C | -0.039 | 0.006 | 8.03E-11 | 0.600583 | 3.174E-05 | 42.24994 |
| rs2286729 | A | G | 0.07 | 0.011 | 1.97E-10 | 0.086402 | 3.042E-05 | 40.49581 |
| rs2364921 | T | C | -0.034 | 0.006 | 1.46E-08 | 0.530998 | 2.412E-05 | 32.11106 |
| rs2388840 | A | G | -0.037 | 0.006 | 6.97E-10 | 0.575311 | 2.857E-05 | 38.02772 |
| rs2389631 | A | C | -0.04 | 0.006 | 2.62E-11 | 0.333824 | 3.339E-05 | 44.44438 |
| rs2431108 | T | C | -0.053 | 0.006 | 1.02E-18 | 0.32836 | 5.862E-05 | 78.02766 |
| rs2598293 | T | C | 0.035 | 0.006 | 5.43E-09 | 0.476808 | 2.556E-05 | 34.02773 |
| rs2838787 | A | G | -0.036 | 0.006 | 1.97E-09 | 0.39209 | 2.705E-05 | 35.99995 |
| rs28552587 | A | G | 0.033 | 0.006 | 3.80E-08 | 0.435629 | 2.273E-05 | 30.24995 |
| rs28582096 | A | G | -0.054 | 0.007 | 1.22E-14 | 0.205367 | 4.471E-05 | 59.51011 |
| rs28611339 | T | G | 0.058 | 0.009 | 1.16E-10 | 0.12895 | 3.12E-05 | 41.5308 |
| rs3131638 | A | G | -0.044 | 0.007 | 3.26E-10 | 0.773994 | 2.968E-05 | 39.51014 |
| rs314281 | T | C | -0.043 | 0.006 | 7.68E-13 | 0.546954 | 3.859E-05 | 51.36103 |
| rs3184470 | A | G | -0.038 | 0.006 | 2.40E-10 | 0.351567 | 3.013E-05 | 40.11105 |
| rs34214423 | A | C | 0.045 | 0.008 | 1.86E-08 | 0.191347 | 2.377E-05 | 31.64058 |
| rs34490907 | C | G | 0.054 | 0.009 | 1.97E-09 | 0.114411 | 2.705E-05 | 35.99995 |
| rs35322724 | A | C | 0.049 | 0.006 | 3.17E-16 | 0.577158 | 5.011E-05 | 66.69434 |
| rs4238755 | A | C | -0.043 | 0.007 | 8.11E-10 | 0.736451 | 2.835E-05 | 37.73464 |
| rs4588900 | A | G | 0.033 | 0.006 | 3.80E-08 | 0.515482 | 2.273E-05 | 30.24995 |
| rs4592425 | T | G | 0.04 | 0.006 | 2.62E-11 | 0.694876 | 3.339E-05 | 44.44438 |
| rs4709655 | T | C | -0.054 | 0.009 | 1.97E-09 | 0.123867 | 2.705E-05 | 35.99995 |
| rs4767645 | T | G | -0.037 | 0.006 | 6.97E-10 | 0.462107 | 2.857E-05 | 38.02772 |
| rs4788203 | A | G | -0.035 | 0.006 | 5.43E-09 | 0.433754 | 2.556E-05 | 34.02773 |
| rs521484 | A | G | -0.04 | 0.007 | 1.10E-08 | 0.766521 | 2.453E-05 | 32.65301 |
| rs524859 | A | G | -0.044 | 0.006 | 2.24E-13 | 0.359842 | 4.04E-05 | 53.7777 |
| rs55772859 | A | C | 0.042 | 0.006 | 2.56E-12 | 0.310843 | 3.681E-05 | 48.99993 |
| rs55972276 | A | C | 0.073 | 0.009 | 5.02E-16 | 0.13676 | 4.943E-05 | 65.79002 |
| rs56097173 | T | C | 0.04 | 0.006 | 2.62E-11 | 0.319651 | 3.339E-05 | 44.44438 |
| rs566673 | T | G | -0.039 | 0.006 | 8.03E-11 | 0.46501 | 3.174E-05 | 42.24994 |
| rs6019663 | T | C | 0.04 | 0.007 | 1.10E-08 | 0.706397 | 2.453E-05 | 32.65301 |
| rs60565673 | T | G | -0.043 | 0.006 | 7.68E-13 | 0.37891 | 3.859E-05 | 51.36103 |
| rs61921611 | T | C | -0.044 | 0.006 | 2.24E-13 | 0.308321 | 4.04E-05 | 53.7777 |
| rs62068188 | T | C | 0.049 | 0.008 | 9.07E-10 | 0.172523 | 2.819E-05 | 37.51557 |
| rs62213452 | T | G | 0.037 | 0.007 | 1.25E-07 | 0.279821 | 2.099E-05 | 27.93873 |
| rs62264767 | A | C | 0.065 | 0.008 | 4.47E-16 | 0.147973 | 4.96E-05 | 66.01553 |
| rs62301574 | C | G | -0.042 | 0.007 | 1.97E-09 | 0.798061 | 2.705E-05 | 35.99995 |
| rs6510033 | A | G | -0.037 | 0.007 | 1.25E-07 | 0.275008 | 2.099E-05 | 27.93873 |
| rs6545798 | A | T | -0.041 | 0.006 | 8.30E-12 | 0.588313 | 3.508E-05 | 46.69437 |
| rs6562066 | T | C | 0.039 | 0.006 | 8.03E-11 | 0.6309 | 3.174E-05 | 42.24994 |
| rs6601080 | A | G | 0.035 | 0.006 | 5.43E-09 | 0.674981 | 2.556E-05 | 34.02773 |
| rs667730 | T | C | 0.033 | 0.006 | 3.80E-08 | 0.42152 | 2.273E-05 | 30.24995 |
| rs6702604 | A | G | -0.037 | 0.006 | 6.97E-10 | 0.416045 | 2.857E-05 | 38.02772 |
| rs671985 | A | G | -0.038 | 0.006 | 2.40E-10 | 0.451658 | 3.013E-05 | 40.11105 |
| rs6756610 | C | G | 0.037 | 0.006 | 6.97E-10 | 0.371982 | 2.857E-05 | 38.02772 |
| rs6808140 | T | C | 0.039 | 0.006 | 8.03E-11 | 0.505785 | 3.174E-05 | 42.24994 |
| rs6888135 | A | C | 0.038 | 0.006 | 2.40E-10 | 0.496301 | 3.013E-05 | 40.11105 |
| rs694786 | T | C | -0.044 | 0.006 | 2.24E-13 | 0.539718 | 4.04E-05 | 53.7777 |
| rs6967168 | T | G | -0.044 | 0.007 | 3.26E-10 | 0.245945 | 2.968E-05 | 39.51014 |
| rs701394 | A | G | -0.036 | 0.006 | 1.97E-09 | 0.362098 | 2.705E-05 | 35.99995 |
| rs7044885 | C | G | -0.041 | 0.006 | 8.30E-12 | 0.558504 | 3.508E-05 | 46.69437 |
| rs715338 | A | G | 0.041 | 0.006 | 8.30E-12 | 0.577291 | 3.508E-05 | 46.69437 |
| rs7214267 | A | G | -0.044 | 0.006 | 2.24E-13 | 0.580876 | 4.04E-05 | 53.7777 |
| rs72657797 | T | C | -0.056 | 0.008 | 2.56E-12 | 0.175601 | 3.681E-05 | 48.99993 |
| rs72773790 | T | C | 0.037 | 0.006 | 6.97E-10 | 0.326845 | 2.857E-05 | 38.02772 |
| rs728017 | A | G | -0.035 | 0.006 | 5.43E-09 | 0.613331 | 2.556E-05 | 34.02773 |
| rs72899452 | T | C | 0.074 | 0.012 | 6.97E-10 | 0.066099 | 2.857E-05 | 38.02772 |
| rs73671843 | A | G | -0.056 | 0.009 | 4.90E-10 | 0.132832 | 2.909E-05 | 38.71599 |
| rs7402939 | T | C | -0.036 | 0.006 | 1.97E-09 | 0.62326 | 2.705E-05 | 35.99995 |
| rs742760 | A | T | 0.043 | 0.008 | 7.66E-08 | 0.185188 | 2.171E-05 | 28.89058 |
| rs7475916 | C | G | -0.037 | 0.006 | 6.97E-10 | 0.645243 | 2.857E-05 | 38.02772 |
| rs7571486 | A | G | -0.039 | 0.007 | 2.53E-08 | 0.250656 | 2.332E-05 | 31.04077 |
| rs7599697 | T | C | -0.037 | 0.006 | 6.97E-10 | 0.357816 | 2.857E-05 | 38.02772 |
| rs76145129 | T | G | -0.05 | 0.009 | 2.77E-08 | 0.125432 | 2.319E-05 | 30.86415 |
| rs7625896 | A | G | 0.036 | 0.006 | 1.97E-09 | 0.34698 | 2.705E-05 | 35.99995 |
| rs8076183 | T | C | -0.038 | 0.006 | 2.40E-10 | 0.448667 | 3.013E-05 | 40.11105 |
| rs8180457 | T | C | -0.056 | 0.008 | 2.56E-12 | 0.839588 | 3.681E-05 | 48.99993 |
| rs8180817 | C | G | -0.049 | 0.006 | 3.17E-16 | 0.431043 | 5.011E-05 | 66.69434 |
| rs823247 | T | C | -0.037 | 0.006 | 6.97E-10 | 0.479707 | 2.857E-05 | 38.02772 |
| rs830716 | C | G | 0.045 | 0.007 | 1.29E-10 | 0.712702 | 3.105E-05 | 41.32647 |
| rs871994 | A | C | 0.035 | 0.006 | 5.43E-09 | 0.435763 | 2.556E-05 | 34.02773 |
| rs908668 | T | C | 0.05 | 0.007 | 9.14E-13 | 0.210534 | 3.833E-05 | 51.02033 |
| rs9394502 | T | C | -0.054 | 0.006 | 2.26E-19 | 0.335881 | 6.085E-05 | 80.99988 |
| rs9527083 | A | G | -0.076 | 0.006 | 9.05E-37 | 0.666794 | 0.0001205 | 160.4442 |
| rs9889282 | A | C | -0.042 | 0.006 | 2.56E-12 | 0.38791 | 3.681E-05 | 48.99993 |
| rs9931543 | T | C | 0.048 | 0.007 | 7.03E-12 | 0.264893 | 3.533E-05 | 47.02034 |

**Table S8. 60 index SNPs represented genetically predicted sleep duration**

| **SNP** | **Effect_allele** | **Other_allele** | **Beta** | **SE** | ***P*** | **EAF** | **R^2^** | **F-statistics** |
| --- | --- | --- | --- | --- | --- | --- | --- | --- |
| rs10173260 | C | T | 0.77 | 0.139 | 3.03E-08 | 0.606336 | 6.88E-05 | 30.68668 |
| rs10421649 | A | T | 0.798 | 0.138 | 7.36E-09 | 0.556369 | 7.49E-05 | 33.43841 |
| rs10483350 | G | A | 1.042 | 0.172 | 1.38E-09 | 0.196427 | 8.23E-05 | 36.70089 |
| rs10761674 | C | T | 0.74 | 0.136 | 5.29E-08 | 0.521702 | 6.64E-05 | 29.60627 |
| rs10973207 | T | G | 1.226 | 0.187 | 5.52E-11 | 0.156772 | 9.63E-05 | 42.98291 |
| rs112230981 | A | G | 1.892 | 0.314 | 1.69E-09 | 0.049549 | 8.14E-05 | 36.30622 |
| rs113113059 | T | C | 0.968 | 0.164 | 3.58E-09 | 0.219862 | 7.81E-05 | 34.83863 |
| rs11567976 | T | C | 0.768 | 0.137 | 2.07E-08 | 0.568315 | 7.04E-05 | 31.42529 |
| rs11602180 | C | T | 1.095 | 0.184 | 2.66E-09 | 0.164484 | 7.94E-05 | 35.41528 |
| rs11621908 | C | T | 1.446 | 0.25 | 7.29E-09 | 0.082793 | 7.5E-05 | 33.45451 |
| rs11643715 | G | C | 0.834 | 0.15 | 2.70E-08 | 0.292681 | 6.93E-05 | 30.91346 |
| rs11885663 | T | C | 0.973 | 0.157 | 5.74E-10 | 0.249784 | 8.61E-05 | 38.40824 |
| rs12246842 | A | G | 0.804 | 0.136 | 3.38E-09 | 0.542162 | 7.83E-05 | 34.94881 |
| rs12567114 | A | G | 0.89 | 0.152 | 4.76E-09 | 0.276388 | 7.68E-05 | 34.28395 |
| rs12607679 | T | C | 1.208 | 0.156 | 9.66E-15 | 0.262435 | 0.000134 | 59.96291 |
| rs1263056 | A | G | 0.768 | 0.137 | 2.07E-08 | 0.482069 | 7.04E-05 | 31.42529 |
| rs13088093 | G | T | 0.976 | 0.144 | 1.22E-11 | 0.336017 | 0.000103 | 45.93807 |
| rs13109404 | T | G | 1.872 | 0.264 | 1.33E-12 | 0.072181 | 0.000113 | 50.28077 |
| rs151014368 | A | G | 0.966 | 0.169 | 1.09E-08 | 0.207268 | 7.32E-05 | 32.67224 |
| rs1517572 | C | A | 0.879 | 0.138 | 1.90E-10 | 0.581239 | 9.09E-05 | 40.57118 |
| rs1553132 | G | A | 0.87 | 0.155 | 1.99E-08 | 0.258654 | 7.06E-05 | 31.50454 |
| rs17427571 | A | G | 0.83 | 0.146 | 1.31E-08 | 0.31517 | 7.24E-05 | 32.3183 |
| rs17732997 | C | G | 0.776 | 0.137 | 1.48E-08 | 0.429775 | 7.19E-05 | 32.0834 |
| rs1776776 | T | C | 1.198 | 0.205 | 5.10E-09 | 0.12657 | 7.65E-05 | 34.15104 |
| rs1939455 | G | T | 1.226 | 0.214 | 1.01E-08 | 0.120323 | 7.36E-05 | 32.82097 |
| rs205024 | T | C | 0.83 | 0.14 | 3.06E-09 | 0.384933 | 7.88E-05 | 35.1478 |
| rs2079070 | C | G | 1.053 | 0.154 | 8.05E-12 | 0.734547 | 0.000105 | 46.75342 |
| rs2192528 | A | G | 0.802 | 0.136 | 3.70E-09 | 0.522413 | 7.79E-05 | 34.77515 |
| rs2231265 | G | A | 0.897 | 0.162 | 3.08E-08 | 0.77159 | 6.87E-05 | 30.65864 |
| rs269054 | A | T | 0.819 | 0.138 | 2.94E-09 | 0.422525 | 7.89E-05 | 35.22149 |
| rs3095508 | C | A | 0.921 | 0.138 | 2.49E-11 | 0.406049 | 9.98E-05 | 44.54092 |
| rs34354917 | C | A | 0.825 | 0.15 | 3.80E-08 | 0.288542 | 6.78E-05 | 30.24986 |
| rs34556183 | A | G | 1.015 | 0.151 | 1.79E-11 | 0.279931 | 0.000101 | 45.18312 |
| rs34731055 | T | C | 1.168 | 0.177 | 4.14E-11 | 0.182129 | 9.76E-05 | 43.54489 |
| rs35531607 | C | T | 0.77 | 0.136 | 1.50E-08 | 0.474151 | 7.18E-05 | 32.05544 |
| rs365663 | A | G | 0.878 | 0.137 | 1.47E-10 | 0.45496 | 9.21E-05 | 41.07201 |
| rs374153 | C | T | 1.057 | 0.186 | 1.33E-08 | 0.842571 | 7.24E-05 | 32.29402 |
| rs4128364 | C | T | 0.876 | 0.143 | 9.02E-10 | 0.339028 | 8.41E-05 | 37.52617 |
| rs4592416 | G | A | 0.881 | 0.136 | 9.30E-11 | 0.461967 | 9.41E-05 | 41.96353 |
| rs4767550 | G | A | 0.858 | 0.139 | 6.71E-10 | 0.413218 | 8.54E-05 | 38.10158 |
| rs55658675 | C | T | 0.788 | 0.142 | 2.87E-08 | 0.352955 | 6.9E-05 | 30.79455 |
| rs56372231 | T | C | 1.017 | 0.144 | 1.64E-12 | 0.332565 | 0.000112 | 49.87868 |
| rs61796569 | T | C | 0.927 | 0.154 | 1.75E-09 | 0.269759 | 8.12E-05 | 36.23398 |
| rs61985058 | T | C | 1.116 | 0.194 | 8.79E-09 | 0.14359 | 7.42E-05 | 33.092 |
| rs62120041 | T | C | 1.567 | 0.274 | 1.07E-08 | 0.066098 | 7.33E-05 | 32.70656 |
| rs6575005 | T | C | 0.934 | 0.159 | 4.25E-09 | 0.242921 | 7.73E-05 | 34.50623 |
| rs7115226 | A | C | 1.594 | 0.261 | 1.01E-09 | 0.073467 | 8.36E-05 | 37.2987 |
| rs72804080 | G | A | 1.068 | 0.192 | 2.66E-08 | 0.148375 | 6.94E-05 | 30.94127 |
| rs73219758 | G | A | 0.984 | 0.15 | 5.38E-11 | 0.291483 | 9.65E-05 | 43.03341 |
| rs75539574 | C | A | 2.175 | 0.244 | 4.93E-19 | 0.085751 | 0.000178 | 79.45787 |
| rs7556815 | A | G | 2.443 | 0.164 | 3.48E-50 | 0.220148 | 0.000497 | 221.9 |
| rs7644809 | T | C | 0.784 | 0.138 | 1.34E-08 | 0.575982 | 7.23E-05 | 32.27543 |
| rs7806045 | T | C | 0.887 | 0.158 | 1.98E-08 | 0.244697 | 7.06E-05 | 31.516 |
| rs7915425 | T | C | 1.144 | 0.179 | 1.65E-10 | 0.823705 | 9.15E-05 | 40.84548 |
| rs8038326 | A | G | 0.955 | 0.152 | 3.32E-10 | 0.273218 | 8.85E-05 | 39.47459 |
| rs915416 | C | G | 1.156 | 0.15 | 1.29E-14 | 0.708933 | 0.000133 | 59.39244 |
| rs9345234 | C | A | 0.781 | 0.138 | 1.52E-08 | 0.577852 | 7.18E-05 | 32.02889 |
| rs9382445 | T | C | 0.872 | 0.14 | 4.71E-10 | 0.375155 | 8.7E-05 | 38.79493 |
| rs9903973 | C | T | 0.766 | 0.136 | 1.78E-08 | 0.534701 | 7.11E-05 | 31.72326 |
| rs9940646 | C | G | 1.017 | 0.137 | 1.14E-13 | 0.423405 | 0.000124 | 55.10599 |

**Table S9. 170 index SNPs represented genetically predicted chronotype**

| **SNP** | **Effect_allele** | **Other_allele** | **Beta** | **SE** | ***P*** | **EAF** | **R^2^** | **F-statistics** |
| --- | --- | --- | --- | --- | --- | --- | --- | --- |
| rs10109566 | A | G | -0.022 | 0.004 | 3.80E-08 | 0.509773 | 4.33E-05 | 30.24991 |
| rs10237162 | T | C | 0.037 | 0.004 | 2.24E-20 | 0.27524 | 0.000123 | 85.56225 |
| rs10254050 | C | G | -0.058 | 0.005 | 4.12E-31 | 0.812194 | 0.000193 | 134.5596 |
| rs1025601 | T | C | -0.022 | 0.004 | 3.80E-08 | 0.383893 | 4.33E-05 | 30.24991 |
| rs10520176 | T | C | 0.038 | 0.004 | 2.10E-21 | 0.500066 | 0.000129 | 90.24974 |
| rs1061032 | T | G | 0.064 | 0.006 | 1.46E-26 | 0.909135 | 0.000163 | 113.7775 |
| rs10742179 | A | G | 0.035 | 0.004 | 2.13E-18 | 0.739087 | 0.00011 | 76.56228 |
| rs10759208 | T | C | -0.025 | 0.004 | 4.10E-10 | 0.390804 | 5.6E-05 | 39.06239 |
| rs10762434 | C | G | 0.025 | 0.004 | 4.10E-10 | 0.776737 | 5.6E-05 | 39.06239 |
| rs10818834 | T | C | 0.03 | 0.004 | 6.38E-14 | 0.268027 | 8.06E-05 | 56.24984 |
| rs10830107 | A | G | 0.028 | 0.005 | 2.14E-08 | 0.204145 | 4.49E-05 | 31.35991 |
| rs10877962 | T | C | 0.036 | 0.004 | 2.26E-19 | 0.412649 | 0.000116 | 80.99977 |
| rs10917513 | T | C | -0.031 | 0.004 | 9.19E-15 | 0.653937 | 8.61E-05 | 60.06233 |
| rs10951325 | T | C | 0.034 | 0.004 | 1.90E-17 | 0.370146 | 0.000104 | 72.24979 |
| rs11032362 | A | G | 0.07 | 0.006 | 1.89E-31 | 0.090903 | 0.000195 | 136.1107 |
| rs11102807 | A | G | -0.022 | 0.004 | 3.80E-08 | 0.462591 | 4.33E-05 | 30.24991 |
| rs11154718 | T | C | -0.023 | 0.004 | 8.92E-09 | 0.572329 | 4.74E-05 | 33.06241 |
| rs11165655 | A | G | -0.028 | 0.004 | 2.56E-12 | 0.525605 | 7.02E-05 | 48.99986 |
| rs11208844 | A | G | -0.029 | 0.005 | 6.63E-09 | 0.138866 | 4.82E-05 | 33.6399 |
| rs1144566 | T | C | 0.231 | 0.011 | 6.56E-98 | 0.970104 | 0.000632 | 440.9987 |
| rs114848860 | A | T | -0.077 | 0.01 | 1.36E-14 | 0.024198 | 8.5E-05 | 59.28983 |
| rs115073088 | A | G | -0.076 | 0.011 | 4.88E-12 | 0.023634 | 6.84E-05 | 47.7354 |
| rs11545787 | A | G | -0.05 | 0.004 | 7.47E-36 | 0.249873 | 0.000224 | 156.2496 |
| rs11588913 | A | G | -0.024 | 0.004 | 1.97E-09 | 0.398975 | 5.16E-05 | 35.9999 |
| rs11611435 | T | C | 0.028 | 0.004 | 2.56E-12 | 0.44706 | 7.02E-05 | 48.99986 |
| rs11670534 | T | C | -0.031 | 0.005 | 5.65E-10 | 0.164748 | 5.51E-05 | 38.43989 |
| rs11677484 | T | G | 0.023 | 0.004 | 8.92E-09 | 0.25496 | 4.74E-05 | 33.06241 |
| rs11845599 | A | G | -0.027 | 0.004 | 1.48E-11 | 0.364579 | 6.53E-05 | 45.56237 |
| rs12040629 | A | G | 0.073 | 0.005 | 2.81E-48 | 0.160286 | 0.000305 | 213.1594 |
| rs12195792 | A | T | 0.034 | 0.004 | 1.90E-17 | 0.268843 | 0.000104 | 72.24979 |
| rs12249410 | T | G | -0.034 | 0.006 | 1.46E-08 | 0.109621 | 4.6E-05 | 32.11102 |
| rs12442008 | T | C | 0.029 | 0.004 | 4.17E-13 | 0.254203 | 7.53E-05 | 52.56235 |
| rs12445235 | C | G | -0.021 | 0.004 | 1.52E-07 | 0.410072 | 3.95E-05 | 27.56242 |
| rs12518401 | A | G | -0.024 | 0.004 | 1.97E-09 | 0.387759 | 5.16E-05 | 35.9999 |
| rs12636669 | T | C | 0.057 | 0.006 | 2.10E-21 | 0.081732 | 0.000129 | 90.24974 |
| rs1278402 | A | G | 0.028 | 0.005 | 2.14E-08 | 0.266291 | 4.49E-05 | 31.35991 |
| rs12808544 | A | C | -0.035 | 0.004 | 2.13E-18 | 0.240317 | 0.00011 | 76.56228 |
| rs12969848 | T | C | 0.036 | 0.004 | 2.26E-19 | 0.529435 | 0.000116 | 80.99977 |
| rs13011556 | C | G | -0.029 | 0.004 | 4.17E-13 | 0.238886 | 7.53E-05 | 52.56235 |
| rs13065394 | T | G | -0.027 | 0.004 | 1.48E-11 | 0.288285 | 6.53E-05 | 45.56237 |
| rs13172141 | A | T | 0.022 | 0.004 | 3.80E-08 | 0.433444 | 4.33E-05 | 30.24991 |
| rs1398346 | T | C | 0.026 | 0.005 | 1.99E-07 | 0.869328 | 3.87E-05 | 27.03992 |
| rs139911 | T | C | -0.034 | 0.004 | 1.90E-17 | 0.575922 | 0.000104 | 72.24979 |
| rs1468945 | A | G | -0.036 | 0.004 | 2.26E-19 | 0.785184 | 0.000116 | 80.99977 |
| rs1502249 | A | G | 0.017 | 0.003 | 1.46E-08 | 0.476136 | 4.6E-05 | 32.11102 |
| rs1508608 | A | G | 0.028 | 0.004 | 2.56E-12 | 0.679012 | 7.02E-05 | 48.99986 |
| rs1559253 | A | G | 0.022 | 0.004 | 3.80E-08 | 0.357233 | 4.33E-05 | 30.24991 |
| rs1599374 | A | G | 0.031 | 0.004 | 9.19E-15 | 0.51312 | 8.61E-05 | 60.06233 |
| rs16939162 | A | G | 0.038 | 0.005 | 2.96E-14 | 0.170261 | 8.28E-05 | 57.75983 |
| rs17396357 | T | C | 0.021 | 0.004 | 1.52E-07 | 0.380199 | 3.95E-05 | 27.56242 |
| rs17448682 | T | C | 0.035 | 0.004 | 2.13E-18 | 0.23187 | 0.00011 | 76.56228 |
| rs17575798 | A | G | -0.034 | 0.004 | 1.90E-17 | 0.19307 | 0.000104 | 72.24979 |
| rs17577073 | A | C | 0.025 | 0.004 | 4.10E-10 | 0.434231 | 5.6E-05 | 39.06239 |
| rs17712705 | A | G | -0.025 | 0.004 | 4.10E-10 | 0.669823 | 5.6E-05 | 39.06239 |
| rs1811899 | T | C | -0.03 | 0.005 | 1.97E-09 | 0.209737 | 5.16E-05 | 35.9999 |
| rs184033703 | A | G | 0.058 | 0.008 | 4.17E-13 | 0.058013 | 7.53E-05 | 52.56235 |
| rs1843888 | A | G | 0.051 | 0.004 | 3.12E-37 | 0.549467 | 0.000233 | 162.562 |
| rs187028 | A | T | -0.022 | 0.003 | 2.24E-13 | 0.683246 | 7.71E-05 | 53.77762 |
| rs1871729 | A | G | -0.023 | 0.004 | 8.92E-09 | 0.31706 | 4.74E-05 | 33.06241 |
| rs1873958 | A | G | 0.028 | 0.004 | 2.56E-12 | 0.407418 | 7.02E-05 | 48.99986 |
| rs1886205 | A | C | 0.029 | 0.004 | 4.17E-13 | 0.759484 | 7.53E-05 | 52.56235 |
| rs1931814 | A | G | 0.026 | 0.004 | 8.03E-11 | 0.52171 | 6.05E-05 | 42.24988 |
| rs2011528 | T | C | -0.033 | 0.005 | 4.11E-11 | 0.830488 | 6.24E-05 | 43.55988 |
| rs2050185 | A | G | 0.022 | 0.004 | 3.80E-08 | 0.626898 | 4.33E-05 | 30.24991 |
| rs2166559 | T | C | -0.033 | 0.005 | 4.11E-11 | 0.140506 | 6.24E-05 | 43.55988 |
| rs2304467 | C | G | -0.024 | 0.004 | 1.97E-09 | 0.398364 | 5.16E-05 | 35.9999 |
| rs2362775 | T | C | -0.022 | 0.004 | 3.80E-08 | 0.471289 | 4.33E-05 | 30.24991 |
| rs2396004 | A | G | 0.021 | 0.004 | 1.52E-07 | 0.565804 | 3.95E-05 | 27.56242 |
| rs2514214 | A | G | 0.027 | 0.005 | 6.66E-08 | 0.731711 | 4.18E-05 | 29.15992 |
| rs2550298 | T | C | -0.04 | 0.004 | 1.52E-23 | 0.377863 | 0.000143 | 99.99971 |
| rs28380327 | A | T | 0.04 | 0.004 | 1.52E-23 | 0.369924 | 0.000143 | 99.99971 |
| rs2844016 | T | C | 0.027 | 0.004 | 1.48E-11 | 0.701844 | 6.53E-05 | 45.56237 |
| rs28458909 | T | C | -0.07 | 0.006 | 1.89E-31 | 0.12244 | 0.000195 | 136.1107 |
| rs28459838 | T | C | 0.027 | 0.004 | 1.48E-11 | 0.765194 | 6.53E-05 | 45.56237 |
| rs2850979 | T | C | -0.023 | 0.004 | 8.92E-09 | 0.759729 | 4.74E-05 | 33.06241 |
| rs2881955 | T | C | 0.027 | 0.004 | 1.48E-11 | 0.278592 | 6.53E-05 | 45.56237 |
| rs2901796 | A | G | 0.025 | 0.004 | 4.10E-10 | 0.603396 | 5.6E-05 | 39.06239 |
| rs2916148 | A | G | 0.028 | 0.004 | 2.56E-12 | 0.456759 | 7.02E-05 | 48.99986 |
| rs2944831 | A | G | 0.025 | 0.004 | 4.10E-10 | 0.297652 | 5.6E-05 | 39.06239 |
| rs308521 | T | C | 0.028 | 0.003 | 1.03E-20 | 0.398016 | 0.000125 | 87.11086 |
| rs3138490 | A | T | 0.024 | 0.004 | 1.97E-09 | 0.517433 | 5.16E-05 | 35.9999 |
| rs34054660 | A | G | 0.025 | 0.005 | 5.73E-07 | 0.425404 | 3.58E-05 | 24.99993 |
| rs35524253 | A | G | 0.034 | 0.004 | 1.90E-17 | 0.356456 | 0.000104 | 72.24979 |
| rs359248 | T | G | -0.028 | 0.003 | 1.03E-20 | 0.550903 | 0.000125 | 87.11086 |
| rs36055559 | A | G | -0.036 | 0.005 | 6.02E-13 | 0.171738 | 7.43E-05 | 51.83985 |
| rs3760381 | A | G | 0.027 | 0.004 | 1.48E-11 | 0.251329 | 6.53E-05 | 45.56237 |
| rs3807651 | A | T | 0.025 | 0.004 | 4.10E-10 | 0.507916 | 5.6E-05 | 39.06239 |
| rs3815983 | T | C | -0.022 | 0.003 | 2.24E-13 | 0.359572 | 7.71E-05 | 53.77762 |
| rs3850174 | A | T | -0.035 | 0.004 | 2.13E-18 | 0.257368 | 0.00011 | 76.56228 |
| rs3867239 | A | G | 0.026 | 0.004 | 8.03E-11 | 0.378754 | 6.05E-05 | 42.24988 |
| rs4121878 | C | G | 0.022 | 0.004 | 3.80E-08 | 0.505416 | 4.33E-05 | 30.24991 |
| rs4241964 | T | G | -0.029 | 0.004 | 4.17E-13 | 0.475895 | 7.53E-05 | 52.56235 |
| rs4269995 | T | C | -0.034 | 0.004 | 1.90E-17 | 0.252184 | 0.000104 | 72.24979 |
| rs4419127 | A | G | 0.044 | 0.004 | 3.82E-28 | 0.334905 | 0.000173 | 120.9997 |
| rs4535583 | T | C | 0.021 | 0.004 | 1.52E-07 | 0.696205 | 3.95E-05 | 27.56242 |
| rs4550384 | T | G | 0.024 | 0.004 | 1.97E-09 | 0.244642 | 5.16E-05 | 35.9999 |
| rs465670 | T | C | 0.024 | 0.004 | 1.97E-09 | 0.542608 | 5.16E-05 | 35.9999 |
| rs4666682 | A | G | -0.025 | 0.004 | 4.10E-10 | 0.176724 | 5.6E-05 | 39.06239 |
| rs4690085 | A | G | -0.019 | 0.003 | 2.40E-10 | 0.469701 | 5.75E-05 | 40.111 |
| rs4698678 | C | G | 0.031 | 0.005 | 5.65E-10 | 0.718347 | 5.51E-05 | 38.43989 |
| rs4800998 | A | T | 0.039 | 0.005 | 6.19E-15 | 0.185154 | 8.72E-05 | 60.83983 |
| rs481214 | A | T | 0.023 | 0.004 | 8.92E-09 | 0.398131 | 4.74E-05 | 33.06241 |
| rs486416 | A | G | -0.02 | 0.003 | 2.62E-11 | 0.636964 | 6.37E-05 | 44.44432 |
| rs4878734 | A | T | 0.022 | 0.004 | 3.80E-08 | 0.482783 | 4.33E-05 | 30.24991 |
| rs4903203 | A | G | 0.025 | 0.004 | 4.10E-10 | 0.677353 | 5.6E-05 | 39.06239 |
| rs495593 | A | G | 0.023 | 0.004 | 8.92E-09 | 0.742164 | 4.74E-05 | 33.06241 |
| rs497338 | T | C | 0.027 | 0.004 | 1.48E-11 | 0.282111 | 6.53E-05 | 45.56237 |
| rs555784 | A | T | -0.025 | 0.004 | 4.10E-10 | 0.382904 | 5.6E-05 | 39.06239 |
| rs55846845 | A | G | -0.021 | 0.003 | 2.56E-12 | 0.52291 | 7.02E-05 | 48.99986 |
| rs57236847 | C | G | 0.027 | 0.005 | 6.66E-08 | 0.396235 | 4.18E-05 | 29.15992 |
| rs58876439 | A | G | 0.047 | 0.007 | 1.89E-11 | 0.069108 | 6.46E-05 | 45.0815 |
| rs6047481 | A | T | 0.025 | 0.004 | 4.10E-10 | 0.327149 | 5.6E-05 | 39.06239 |
| rs6131805 | T | G | 0.026 | 0.004 | 8.03E-11 | 0.598865 | 6.05E-05 | 42.24988 |
| rs6131942 | A | G | -0.026 | 0.003 | 4.45E-18 | 0.579734 | 0.000108 | 75.1109 |
| rs61875203 | T | C | 0.026 | 0.004 | 8.03E-11 | 0.277065 | 6.05E-05 | 42.24988 |
| rs61990287 | A | C | 0.025 | 0.004 | 4.10E-10 | 0.28056 | 5.6E-05 | 39.06239 |
| rs62082402 | T | G | 0.05 | 0.006 | 7.86E-17 | 0.191615 | 9.95E-05 | 69.44425 |
| rs62182135 | A | C | -0.024 | 0.003 | 1.24E-15 | 0.330662 | 9.17E-05 | 63.99982 |
| rs6429233 | A | G | 0.02 | 0.004 | 5.73E-07 | 0.452758 | 3.58E-05 | 24.99993 |
| rs6477309 | T | C | 0.031 | 0.004 | 9.19E-15 | 0.665763 | 8.61E-05 | 60.06233 |
| rs6560218 | T | C | -0.022 | 0.004 | 3.80E-08 | 0.516125 | 4.33E-05 | 30.24991 |
| rs662094 | A | G | 0.028 | 0.004 | 2.56E-12 | 0.495966 | 7.02E-05 | 48.99986 |
| rs66617308 | T | C | 0.018 | 0.003 | 1.97E-09 | 0.328713 | 5.16E-05 | 35.9999 |
| rs6727752 | A | G | 0.026 | 0.004 | 8.03E-11 | 0.372664 | 6.05E-05 | 42.24988 |
| rs6794796 | A | G | 0.025 | 0.004 | 4.10E-10 | 0.712127 | 5.6E-05 | 39.06239 |
| rs67988891 | C | G | -0.036 | 0.004 | 2.26E-19 | 0.318902 | 0.000116 | 80.99977 |
| rs6838677 | A | C | -0.021 | 0.004 | 1.52E-07 | 0.330694 | 3.95E-05 | 27.56242 |
| rs6846730 | T | C | -0.032 | 0.004 | 1.24E-15 | 0.232489 | 9.17E-05 | 63.99982 |
| rs6958557 | T | G | 0.026 | 0.004 | 8.03E-11 | 0.392993 | 6.05E-05 | 42.24988 |
| rs6967481 | T | C | 0.032 | 0.003 | 1.46E-26 | 0.496898 | 0.000163 | 113.7775 |
| rs6968240 | A | C | 0.022 | 0.003 | 2.24E-13 | 0.426545 | 7.71E-05 | 53.77762 |
| rs6988733 | T | C | 0.023 | 0.004 | 8.92E-09 | 0.347971 | 4.74E-05 | 33.06241 |
| rs6993892 | T | C | -0.035 | 0.004 | 2.13E-18 | 0.390081 | 0.00011 | 76.56228 |
| rs7111582 | A | G | -0.039 | 0.005 | 6.19E-15 | 0.895195 | 8.72E-05 | 60.83983 |
| rs7143933 | T | G | 0.025 | 0.004 | 4.10E-10 | 0.737526 | 5.6E-05 | 39.06239 |
| rs7203707 | A | C | -0.02 | 0.003 | 2.62E-11 | 0.51713 | 6.37E-05 | 44.44432 |
| rs7248205 | T | C | 0.027 | 0.004 | 1.48E-11 | 0.600265 | 6.53E-05 | 45.56237 |
| rs72729847 | T | C | -0.03 | 0.005 | 1.97E-09 | 0.196931 | 5.16E-05 | 35.9999 |
| rs72790386 | T | G | 0.06 | 0.011 | 4.91E-08 | 0.03297 | 4.26E-05 | 29.75198 |
| rs72829706 | A | G | 0.056 | 0.009 | 4.90E-10 | 0.039466 | 5.55E-05 | 38.71594 |
| rs72841368 | A | T | -0.03 | 0.005 | 1.97E-09 | 0.18778 | 5.16E-05 | 35.9999 |
| rs72950188 | T | C | 0.045 | 0.007 | 1.29E-10 | 0.075231 | 5.92E-05 | 41.32641 |
| rs73026775 | A | G | -0.034 | 0.006 | 1.46E-08 | 0.127364 | 4.6E-05 | 32.11102 |
| rs7304278 | A | G | -0.029 | 0.004 | 4.17E-13 | 0.723736 | 7.53E-05 | 52.56235 |
| rs73050286 | T | C | 0.03 | 0.004 | 6.38E-14 | 0.21685 | 8.06E-05 | 56.24984 |
| rs7429614 | T | G | 0.035 | 0.004 | 2.13E-18 | 0.418321 | 0.00011 | 76.56228 |
| rs747003 | T | C | 0.02 | 0.004 | 5.73E-07 | 0.392968 | 3.58E-05 | 24.99993 |
| rs76064513 | T | C | 0.034 | 0.006 | 1.46E-08 | 0.130821 | 4.6E-05 | 32.11102 |
| rs7700110 | A | G | 0.024 | 0.004 | 1.97E-09 | 0.258118 | 5.16E-05 | 35.9999 |
| rs7701529 | A | T | -0.03 | 0.004 | 6.38E-14 | 0.761786 | 8.06E-05 | 56.24984 |
| rs7721608 | T | G | 0.02 | 0.003 | 2.62E-11 | 0.464207 | 6.37E-05 | 44.44432 |
| rs7845620 | A | C | -0.043 | 0.005 | 7.97E-18 | 0.164811 | 0.000106 | 73.95979 |
| rs7943634 | T | C | -0.024 | 0.004 | 1.97E-09 | 0.308716 | 5.16E-05 | 35.9999 |
| rs7959983 | T | C | -0.03 | 0.004 | 6.38E-14 | 0.405035 | 8.06E-05 | 56.24984 |
| rs80097534 | T | G | -0.036 | 0.006 | 1.97E-09 | 0.098262 | 5.16E-05 | 35.9999 |
| rs80271258 | T | C | -0.089 | 0.006 | 8.92E-50 | 0.086385 | 0.000315 | 220.0271 |
| rs909757 | T | C | 0.02 | 0.003 | 2.62E-11 | 0.369335 | 6.37E-05 | 44.44432 |
| rs9347926 | A | T | 0.026 | 0.004 | 8.03E-11 | 0.553843 | 6.05E-05 | 42.24988 |
| rs9381812 | A | G | -0.05 | 0.004 | 7.47E-36 | 0.294213 | 0.000224 | 156.2496 |
| rs938836 | A | G | -0.021 | 0.003 | 2.56E-12 | 0.465659 | 7.02E-05 | 48.99986 |
| rs9416744 | A | C | 0.034 | 0.004 | 1.90E-17 | 0.743157 | 0.000104 | 72.24979 |
| rs9436119 | A | G | 0.04 | 0.003 | 1.48E-40 | 0.395193 | 0.000255 | 177.7773 |
| rs9479402 | T | C | -0.219 | 0.018 | 4.68E-34 | 0.0108 | 0.000212 | 148.0274 |
| rs9558942 | T | C | -0.019 | 0.004 | 2.03E-06 | 0.326647 | 3.23E-05 | 22.56244 |
| rs9573980 | A | G | 0.127 | 0.01 | 5.91E-37 | 0.033732 | 0.000231 | 161.2895 |
| rs9597241 | A | C | 0.033 | 0.004 | 1.58E-16 | 0.188367 | 9.75E-05 | 68.0623 |
| rs9636202 | A | G | -0.026 | 0.004 | 8.03E-11 | 0.267104 | 6.05E-05 | 42.24988 |
| rs9664044 | T | C | -0.027 | 0.004 | 1.48E-11 | 0.23287 | 6.53E-05 | 45.56237 |
| rs9836621 | T | C | -0.028 | 0.004 | 2.56E-12 | 0.518868 | 7.02E-05 | 48.99986 |
| rs9997394 | A | G | -0.025 | 0.004 | 4.10E-10 | 0.290118 | 5.6E-05 | 39.06239 |

**Table S10. 3 index SNPs represented genetically predicted disorder of the sleep-wake schedule**

| **SNP** | **Effect_allele** | **Other_allele** | **Beta** | **SE** | ***P*** | **R^2^** | **F-statistics** |
| --- | --- | --- | --- | --- | --- | --- | --- |
| rs72792649 | G | A | 0.827 | 0.155 | 9.30E-08 | 7.67E-05 | 28.51500116 |
| rs34751835 | C | T | 0.554 | 0.106 | 1.62E-07 | 7.39E-05 | 27.44569407 |
| rs4501174 | C | T | 0.904 | 0.176 | 2.81E-07 | 7.10E-05 | 26.37523018 |

| **Exclude palindromic SNPs** | **Method** | **NSNP** | **Beta** | **SE** | ***P*** | **Q_pval** | **Outlier** | **Pleiotropy** |
| --- | --- | --- | --- | --- | --- | --- | --- | --- |
| rs11012732，rs56194509 | MR Egger | 8 | 0.235479 | 0.9908093 | 0.9773012 | 0.008016315 |  | 0.2378866 |
|  | Weighted median | 8 | 0.834209 | 1.0002805 | 0.9976566 |  |  |  |
|  | Inverse variance weighted | 8 | 0.8592445 | 0.9996994 | 0.9963836 | 0.001498758 |  |  |

**Table S11. MR results of APA on myopia**

**Table S12. MR results of insomnia on myopia**

| **Exclude palindromic SNPs** | **Method** | **NSNP** | **Beta** | **SE** | ***P*** | **Q_pval** | **Outlier** | **Pleiotropy** |
| --- | --- | --- | --- | --- | --- | --- | --- | --- |
| rs11001276, rs11756035, rs12030482, rs12991815, rs214934, rs2216427, rs2221119, rs34490907, rs62301574, rs6545798, rs6756610, rs7044885, rs742760, rs7475916, rs8180817, rs830716 | MR Egger | 103 | -0.019635838 | 0.010069217 | 0.05393811 | 6.063112e-08 | rs11588755，rs6702604 | | 0.06362551 | | --- | |  | | |  | | --- | | |
|  | Weighted median | 103 | 0.001088387 | 0.002168298 | 0.61569955 |  |  |  |
|  | Inverse variance weighted | 103 | -0.001132777 | 0.002036589 | 0.57806525 | 1.534174e-08 |  |  |

**Table S13. MR results of sleep duration on myopia**

| **Exclude palindromic SNPs** | **Method** | **NSNP** | **Beta** | **SE** | ***P*** | **Q_pval** | **Outlier** | **Pleiotropy** |
| --- | --- | --- | --- | --- | --- | --- | --- | --- |
| rs10421649, rs11643715, rs17732997, rs2079070, rs269054, rs915416, rs9940646 | MR Egger | 53 | 6.29655E-05 | 0.000469291 | 0.8937949 | 6.374046e-07 | rs11602180，rs2231265，rs75539574 | | 0.8379462 | | --- | |  | | |  | | --- | | |
|  | Weighted median | 53 | -4.04986E-06 | 0.000153579 | 0.9789623 |  |  |  |
|  | Inverse variance weighted | 53 | -2.94427E-05 | 0.000133516 | 0.8254674 | 9.513806e-07 |  |  |

**Table S14. MR results of chronotype on myopia**

| **Exclude palindromic SNPs** | **Method** | **NSNP** | **Beta** | **SE** | ***P*** | **Q_pval** | **Outlier** | **Pleiotropy** |
| --- | --- | --- | --- | --- | --- | --- | --- | --- |
| rs10254050, rs10762434, rs114848860, rs12195792, rs12445235, rs13011556, rs13172141, rs187028, rs2304467, rs28380327, rs3138490, rs3807651, rs3850174, rs4121878, rs4698678, rs4800998, rs481214, rs4878734, rs555784, rs57236847, rs6047481, rs67988891, rs72841368, rs7701529, rs9347926 | MR Egger | 145 | 0.002865328 | 0.00556976 | 0.607736 | 6.574207e-07 | rs1811899，rs35524253 | | 0.6561464 | | --- | |  | | |  | | --- | | |
|  | Weighted median | 145 | 0.000369971 | 0.002852984 | 0.8968206 |  |  |  |
|  | Inverse variance weighted | 145 | 0.000576665 | 0.002164242 | 0.7898918 | 8.031937e-07 |  |  |

**Table S15. MR results of disorder of the sleep-wake schedule on myopia**

| **Exclude palindromic SNPs** | **Method** | **NSNP** | **Beta** | **SE** | **P** | **Q_pval** | **Outlier** |
| --- | --- | --- | --- | --- | --- | --- | --- |
| -- | MR Egger | 3 | 0.001160981 | 0.00102149 | 0.2557238 | 0.5306616 | -- |
|  | Weighted median | 3 | -0.001167063 | 0.004604715 | 0.8419765 |  |  |
|  | Inverse variance weighted | 3 | 0.001588071 | 0.001200005 | 0.1857068 | 0.7182182 |  |


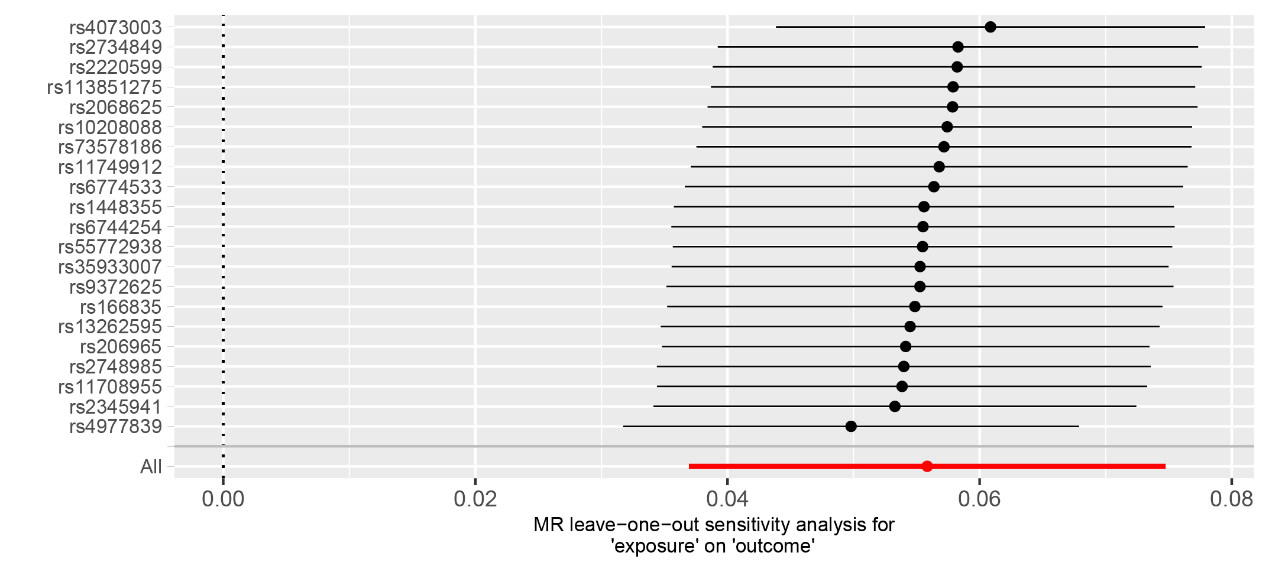


**Figure S1. Leave-one-out analysis from genetically predicted t computer use on myopia**


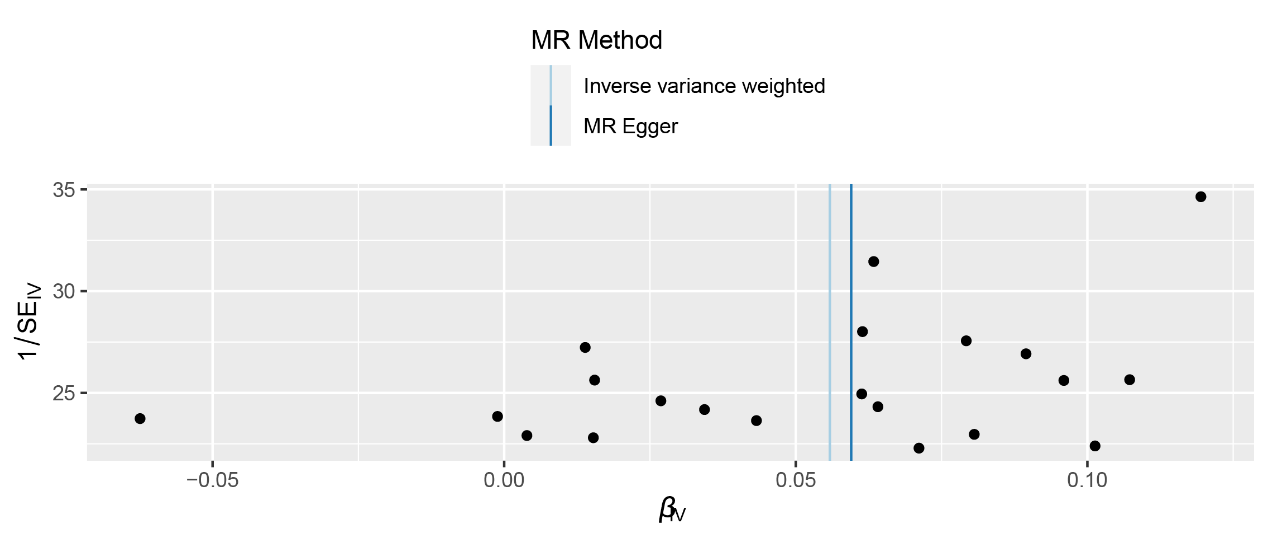


**Figure S2. Funnel plot from genetically predicted t computer use on myopia**


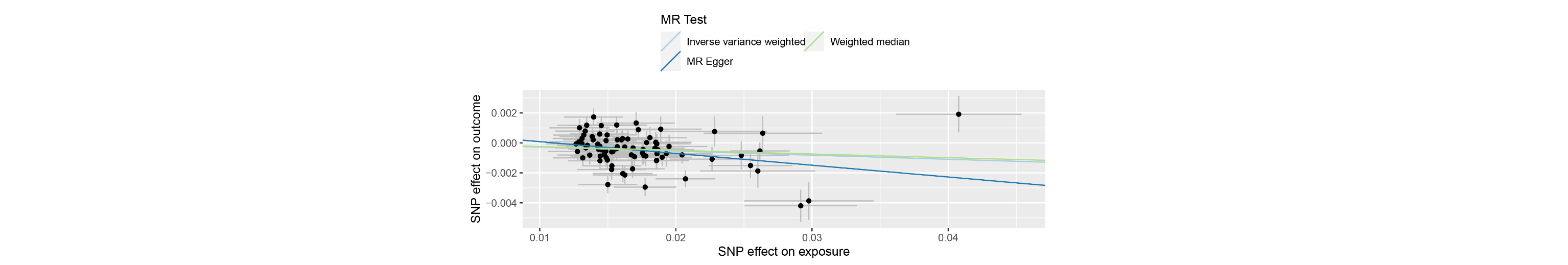


**Figure S3. Scatter plot of SNPs associated with television watching and their risk of myopia after outliers removal with MR-PRESSO.**


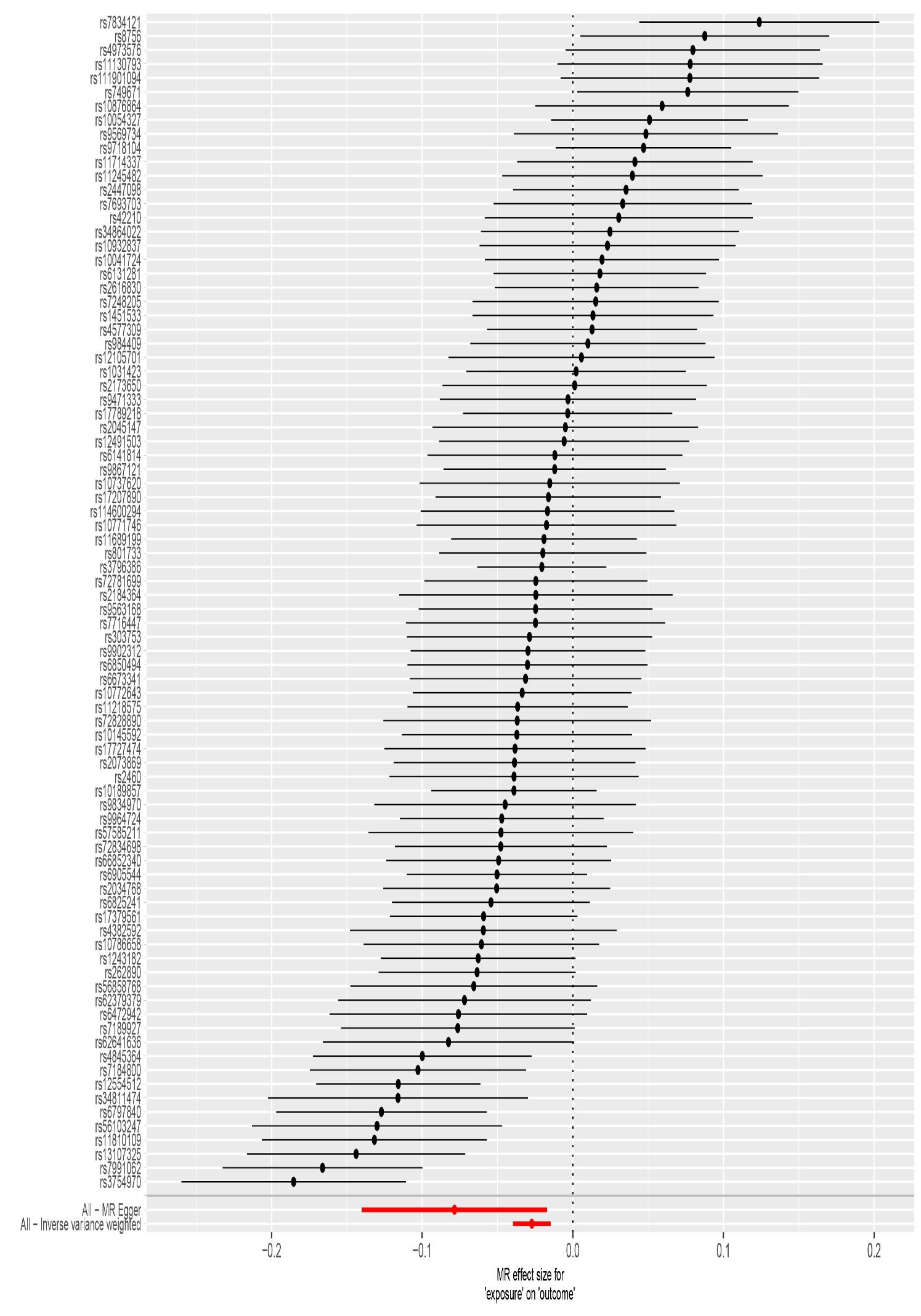


**Figure S4. Forest plot of SNPs associated with television watching and their risk of myopia after outliers removal with MR-PRESSO.**


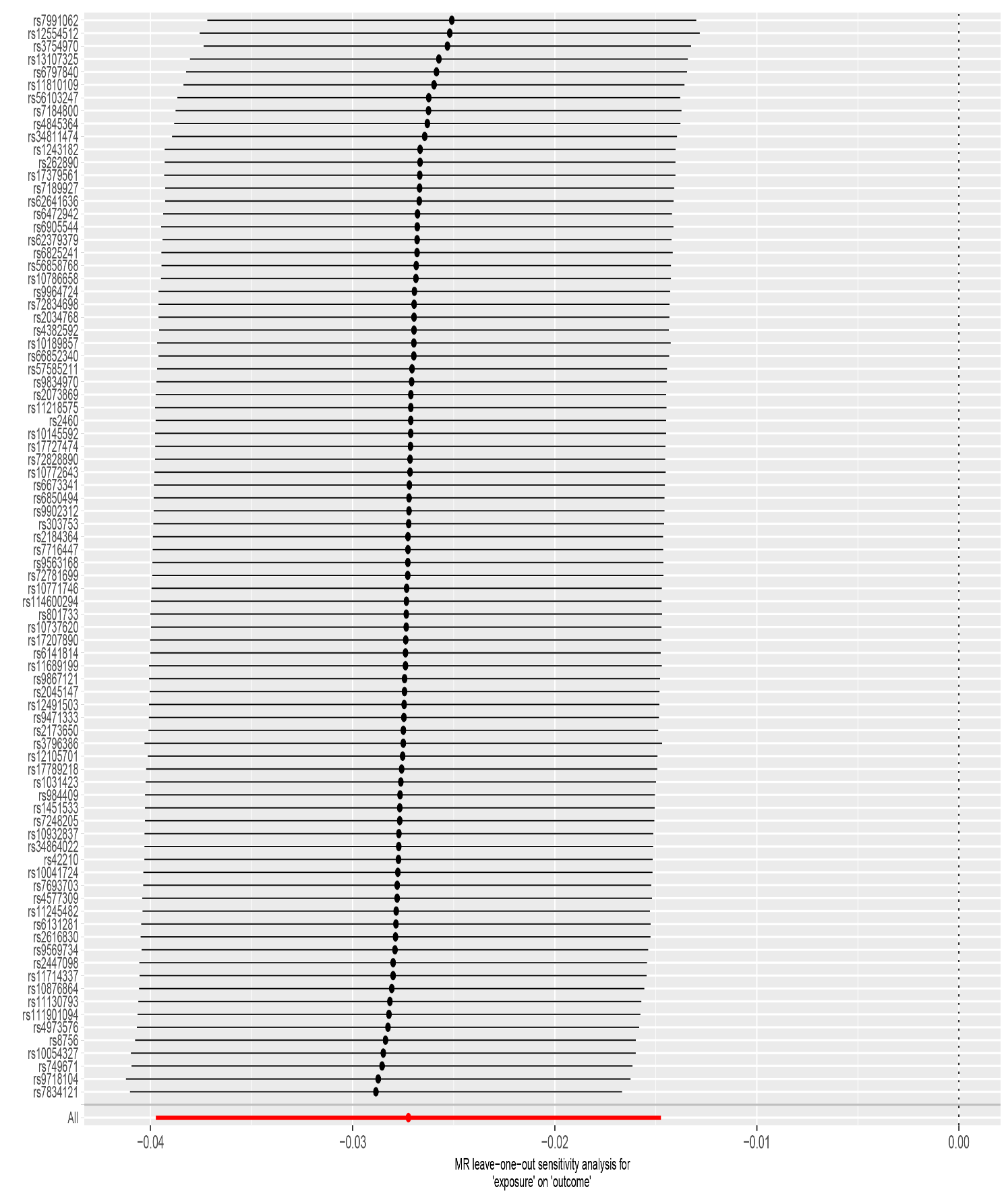


**Figure S5. Leave-one-out of SNPs associated with television watching and their risk of myopia after outliers removal with MR-PRESSO.**


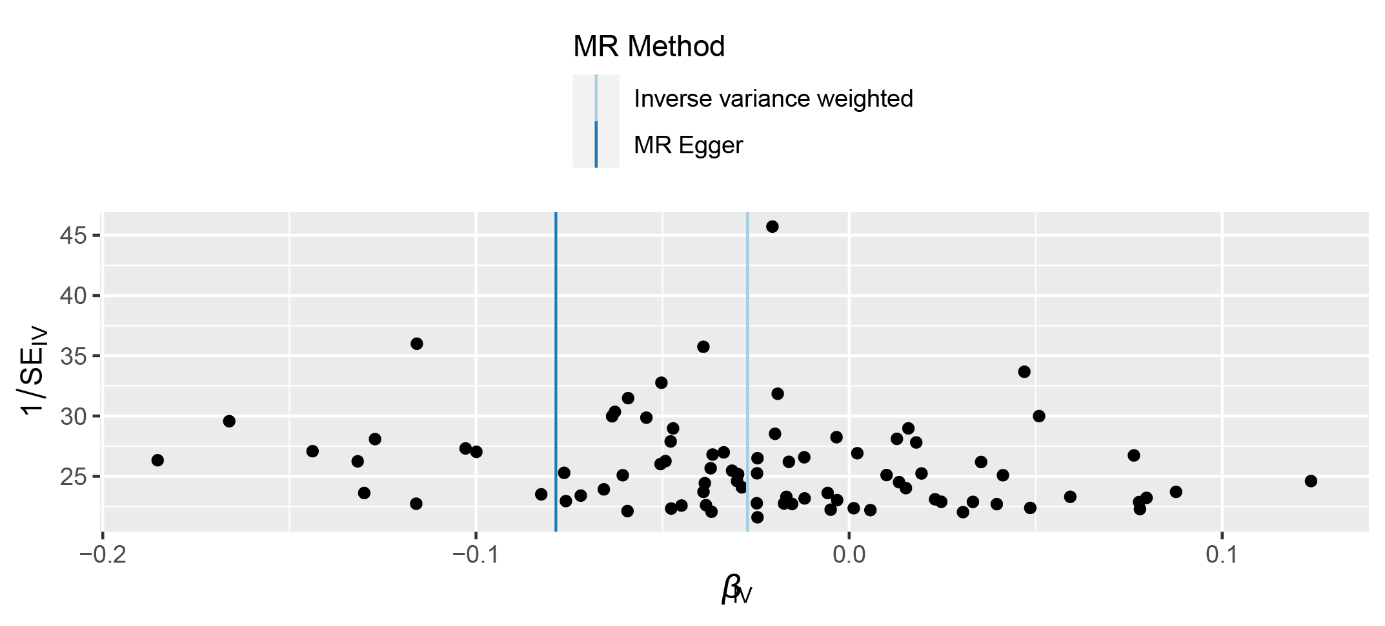


**Figure S6. Funnel plot of SNPs associated with television watching and their risk of myopia after outliers removal with MR-PRESSO.**


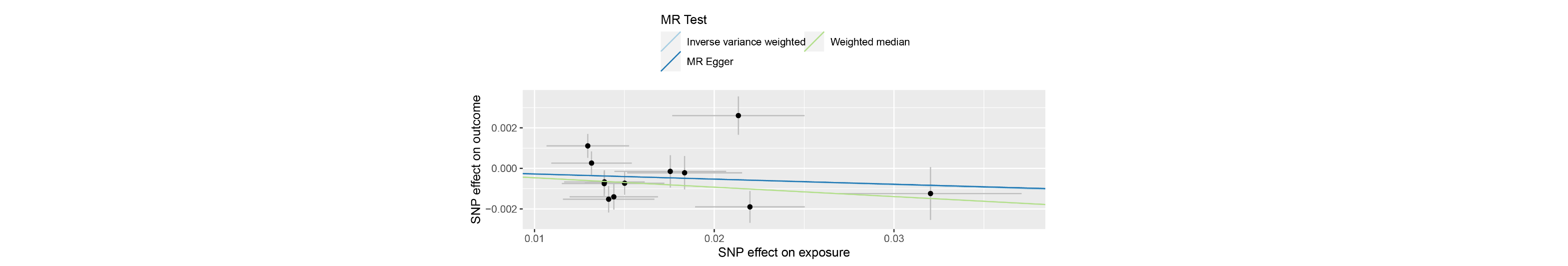


**Figure S7. Scatter plot of SNPs associated with MVPA and their risk of myopia after tightening instrument P value threshold and outliers removal with MR-PRESSO.**


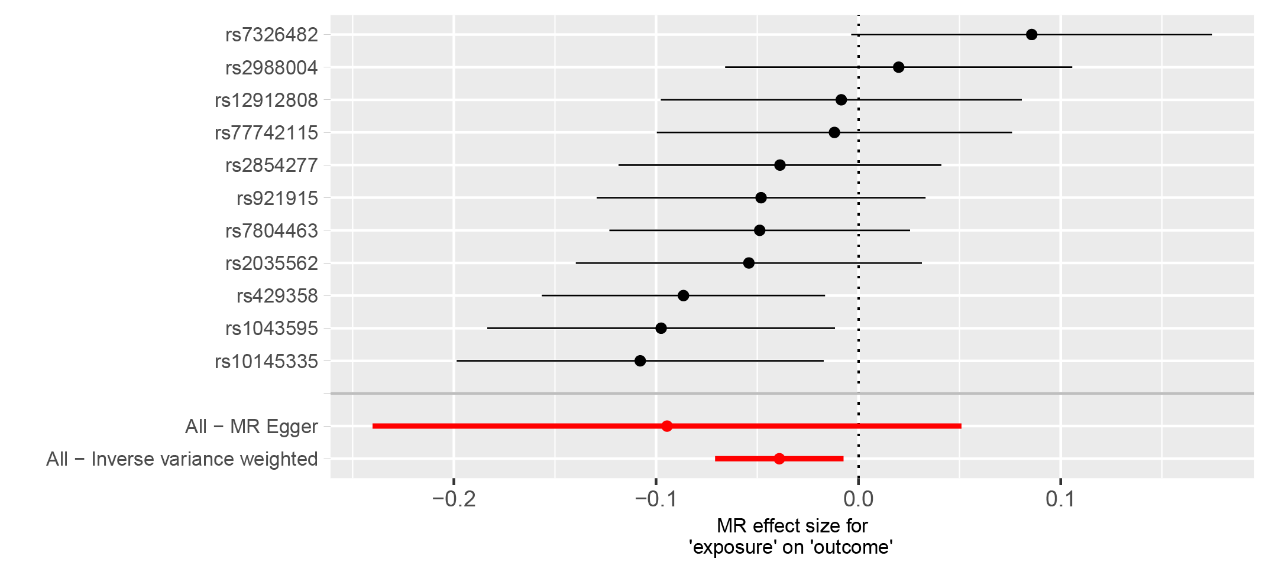


**Figure S8. Forest plot of SNPs associated with MVPA and their risk of myopia after tightening instrument P value threshold and outliers removal with MR-PRESSO.**


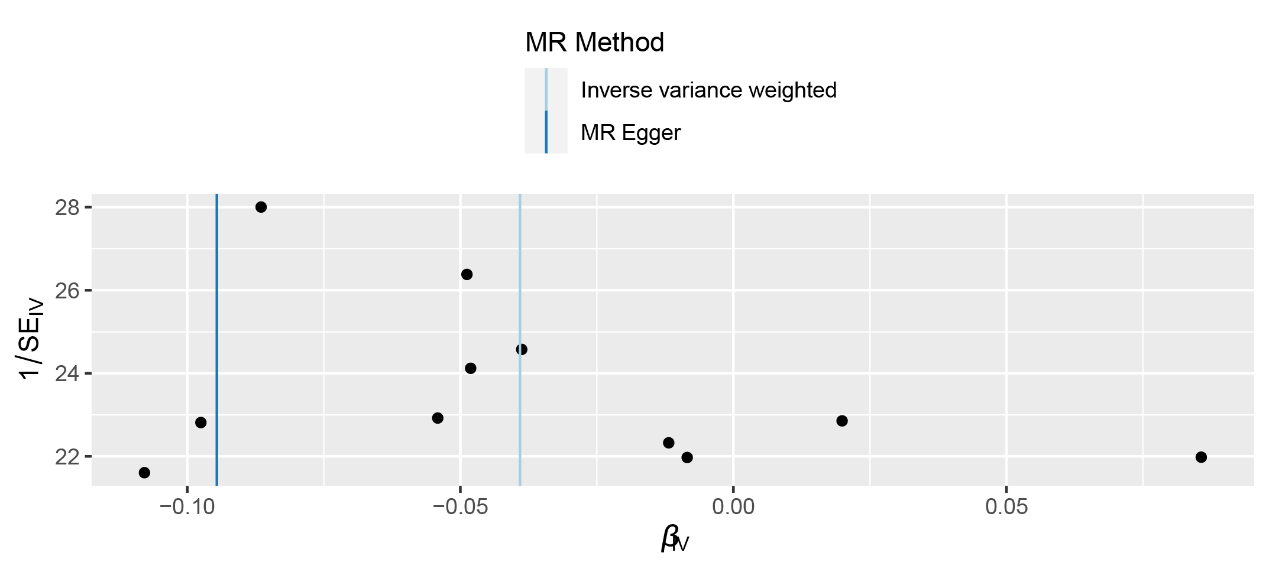


**Figure S9. Funnel plot of SNPs associated with MVPA and their risk of myopia after tightening instrument P value threshold and outliers removal with MR-PRESSO.**


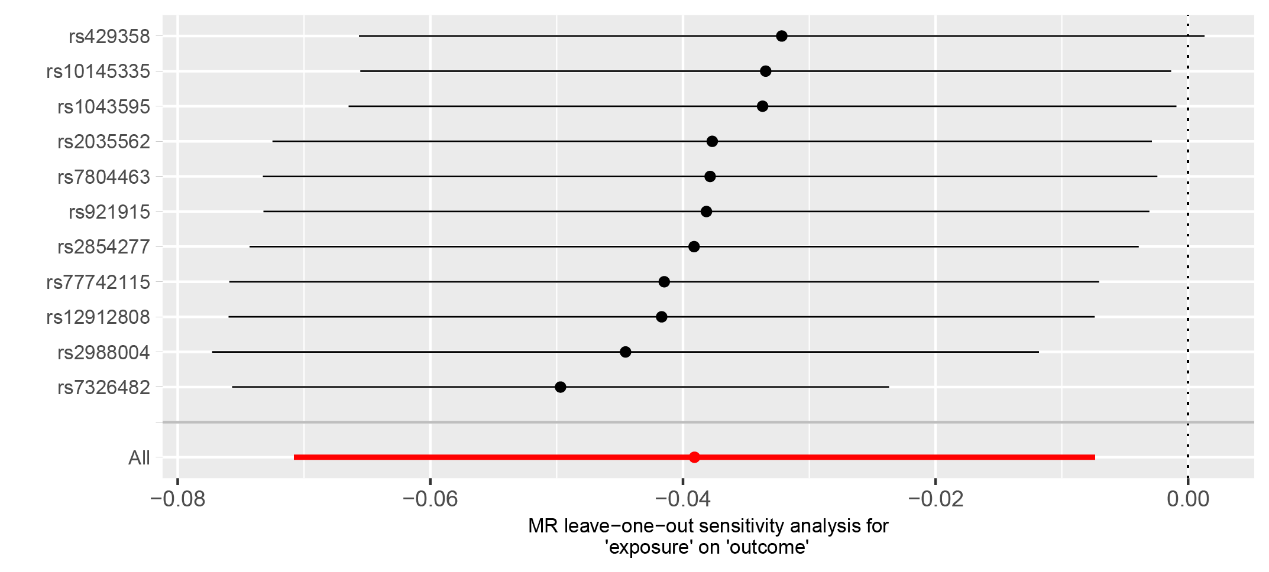


**Figure S10. Leave-one-out of SNPs associated with MVPA and their risk of myopia after tightening instrument P value threshold and outliers removal with MR-PRESSO.**
